# *Punica granatum* extract demonstrates antiparasitic effects against *Caligus clemensi* through in silico and in vitro studies

**DOI:** 10.1038/s41598-025-19529-y

**Published:** 2025-10-07

**Authors:** Marwa M. Attia, Mohamed Abdelsalam, Hend I. Mohamed, Azizeh Shadidizaji, Asmaa W. Soliman, Mohamad Warda

**Affiliations:** 1 Department of Parasitology, Faculty of Veterinary Medicine, Cairo University, Giza, Egypt; 2 Department of Aquatic Animal Medicine and Management, Faculty of Veterinary Medicine, Cairo University, Giza, Egypt; 3 Department of Parasitology, Faculty of Veterinary Medicine, Beni-Suef University, Beni-Suef, 62511 Egypt; 4 Vaccine Development Application and Research Center, Atatürk University, Erzurum, Turkey; 5 National Institute of Oceanography and Fisheries (NIOF), Cairo, Egypt; 6 Department of Physiology, Faculty of Veterinary Medicine, Atatürk University, Erzurum, Turkey; 7 Department of Biochemistry, Faculty of Veterinary Medicine, Cairo University, Giza, Egypt

**Keywords:** *Punica granatum*, *Caligus clemensi*, In silico study, Antiparasitic activity, GC‒MS analysis, Biological techniques, Computational biology and bioinformatics, Ecology

## Abstract

The increasing resistance of aquatic ectoparasites, particularly *Caligus clemensi*, to synthetic antiparasitic agents highlights the need for exploration of alternative competing strategies in aquaculture. This study assessed the antiparasitic potential of *Punica granatum* (pomegranate) methanolic extract, focusing on its bioactive phytochemicals and their synergistic effects. Using gas chromatography‒mass spectrometry (GC‒MS) analysis conducted in this study, we identified a diverse range of compounds, including monoterpenes, aromatic hydrocarbons, and fatty acid derivatives, many of which are known for their neurotoxic, membrane-disrupting, and antimicrobial activities. Notable compounds, such as α-terpinene, γ-terpinene, δ-3-carene, and terpineol, were found to be major constituents of the extract. The presence of *p*-cymene and *m*-cymene in the extract may contribute synergistically to its antiparasitic activity by enhancing parasite cell membrane permeability and facilitating the uptake of other bioactive constituents. In addition, in silico molecular docking analysis of a representative pomegranate-derived compound (1-methyl-4-(1-methylethylidene)cyclohexene) revealed strong binding affinity to key detoxification enzymes such as glutathione S-transferase theta 1–1 (GSTT1) and cytochrome P450 3A24. These interactions suggest potential inhibitory effects, which may impair the parasite’s detoxification pathways and metabolic resistance mechanisms. The observed antiparasitic activity is likely due to a novel multimodal mechanism involving neurotoxic, membrane-disruptive, and enzyme-inhibitory actions. *P. granatum* extract offers a sustainable, eco-friendly alternative to synthetic agents, with reduced resistance risk. This is the first report combining phytochemical profiling, molecular docking, and bioassays to demonstrate its efficacy against *C. clemensi*. These findings support its potential as a natural, broad-spectrum parasiticide for sustainable aquaculture and justify further in vivo and toxicological studies.

**Supplementary Information:**

The online version contains supplementary material available at 10.1038/s41598-025-19529-y.

## Introduction

The aquaculture sector currently contributes approximately half of the global fish supply, playing a significant role alongside capture fisheries and in the domestication of new fish species^1^. In many tropical and subtropical regions, where malnutrition is widespread, fish serve not only as a vital source of animal protein but also as an important commodity for trade and income generation^2^. Food insecurity and nutrient deficiency, however, are their major challenges in developing nations^3,4^, where the population continues to rise with the demand for fish as an alternative high-quality protein source increase. Mullets are considered essential food resources and support the livelihoods of many communities through both capture fisheries and aquaculture^5^. However, fish are vulnerable to a wide range of diseases, including parasitic infections. Parasitic diseases account for approximately 80% of fish diseases, particularly in warm-water species^5^.

Parasitic infections significantly disrupt fish physiology, leading to the secretion of proteolytic enzymes, immunosuppression, and damage to host tissues and organs^2,6^. These infections, both during and following outbreaks, impede growth performance, increase susceptibility to predation, compete with the host for nutrients, and result in nutrient depletion. Consequently, they contribute to substantial economic losses through reduced market value, increased treatment expenditures, and elevated mortality rates^7^. Ectoparasitic infestations are associated with diminished farm productivity and operational efficiency, with cascading effects on farmers’ livelihoods, employment rates, and regional food security^8^. Among the most pathogenic ectoparasites are parasitic crustaceans, which possess a direct life cycle and actively feed on host blood and tissues, particularly targeting the gills and fins, resulting in significant physiological stress and morbidity^9–11^.

Copepods are common crustacean parasites that infest fresh, cultured, and wild marine fish^9–11^. Members of Caligidae are considered the most often reported species that infect marine fish, known as sea lice, and include 28 genera and more than 400 species^12^. The genus *Caligus* is the most common species within Caligidae and is characterized by a tubular mouth, flat mandibles, and long blades with distal ends carrying a row of teeth on one margin^13^. Most *Caligus* species exclusively attack marine environments, and some species may infect brackish water fishes^14^. In Egypt, such parasites were obtained from the mullet fish *Mugil cephalus (M. cephalus)*^11^. They attach to the body surface and gill cavities of the fish^15^. While *Caligus* infections may not cause immediate massive mortalities in *M. cephalus* under normal circumstances, they significantly reduce growth rates and market value^9–11^. However, severe infestations combined with secondary infections can lead to mortality rates reaching 50%. Infected fish suffer from emaciation, dark-colored bodies, and deep damage, with skin loss and sloughing. Respiratory manifestations include great mucous secretions and significant damage to the gills^11,16^. Recent studies have documented persistent sea lice infestations on wild juvenile salmon despite aquaculture production cessation^17^, highlighting the complex ecology of these parasites. Advanced monitoring techniques, including environmental DNA detection, have enhanced our understanding of sea lice population dynamics^18,19^.

The prevention of parasite transmission from aquaculture facilities to wild fish stocks (backspill) requires the implementation of control programs to protect native fish populations^20,21^. Chemotherapeutants and medical products may be the farmer’s primary and handiest choice, but physical, biological, immunoprophylactic, and hereditary control strategies are obtainable as long-lasting alternatives^22^. The application of management rules for fish ectoparasites is essential for contemporary fish culture. Protozoan infection, monogenetic trematodes, copepods, and leeches can infect fish in hatcheries or rearing ponds^11,22^. Unless controlled, these parasites may cause losses either leading to the death of fish or exposing them to secondary bacterial or fungal infections. For many years, the preferred chemical for ectoparasite treatment has been formalin. The application of chemicals for treatment has many drawbacks^9^. Formalin at a 250 ppm level has been shown to be dangerous to fish when left for an hour; heavily parasitized fish are frequently killed, and lesser amounts frequently provide only modest control^17^. When formalin is applied under hot conditions, it commonly causes decreased oxygen levels and fish loss^19^.

Although challenges with the use of formalin have not prevented its use, workers need other beneficial ectoparasite controls. Malachite green, copper sulfate, and potassium permanganate have been applied to ponds, but all have restricted efficiency, are toxic under fluctuating pH or water hardness conditions, or are toxic to specific species^23,24^. Sodium chloride, potassium dichromate, PMA, and acriflavine were used. These chemicals have constraints on their use^19^. In fish farming, chemicals (e.g., formalin, sodium chloride, acetic acid, potassium permanganate, copper sulfate, praziquantel, parathion, levamisole, fenbendazole, ivermectin, emamectin benzoate, diflubenzuron, etc.) are widely used. However, their use might present challenges such as resistance and discrimination toward the environment and humans because of the accumulation of toxic residues, so chemical application is often restricted in many countries^25^. Furthermore, anthelmintics are potentially very costly^26,27^.

Such problems encourage the use of medicinal plants as substitutes for farmed fish^28–32^. The use of natural plants for the treatment of fish parasites has widely increased. Herbal therapy is a potentially effective alternative for fish farming, as it may be less expensive and more effective than chemotherapy^28,30–32^. Systematic reviews have established the growing importance of plant-derived compounds as sustainable alternatives in aquaculture disease management^24–32^. Worldwide, improvements in disease management may result in increased financial viability, increased incomes for farmers, and increased benefits of fish farming to local, regional, and national economies^33^.

*Punica granatum* (pomegranate) was among the first edible fruits identified and has long been used in conventional medicine in America, Asia, Africa, and Europe. Pomegranates have been used to treat inflammation because they have high antioxidant capacity. It has several health benefits and protects against several diseases^34^. Moreover, pomegranate is used as an antiparasitic mediator^35^. Several studies have revealed that pomegranate extract has antiparasitic effects on intestinal cestodes and trematodes^36^ and has antiprotozoan efficacy^37,38^. Recently, pomegranate has been shown to be effective against *Cryptosporidium parvum* and to have an anticoccidial effect^39^. Previous phytochemical analyses of pomegranate have revealed a chemically diverse profile dominated by monoterpenes, aromatic hydrocarbons, and fatty acid derivatives—classes known for potent antiparasitic, antimicrobial, and anti-inflammatory activities^40^. Monoterpenes are particularly prominent in pomegranate extracts, with their lipophilic nature allowing them to integrate into and disrupt lipid bilayers, contributing to their efficacy against ectoparasites^41^. Key monoterpenes previously identified include α-terpinene, γ-terpinene, δ-3-carene, and terpineol, which are associated with well-documented biological activities^42^. α-Terpinene exerts antioxidant and insecticidal effects by inducing oxidative stress and increasing membrane permeability, leading to neuromuscular dysfunction in parasites^43^. γ-Terpinene likely exerts a synergistic effect by enhancing membrane penetration of other toxic compounds^44^. δ-3-Carene, a bicyclic monoterpene, has neurotoxic properties that disrupt ion channels and neurotransmission, causing paralysis and feeding inhibition in parasites^45^. Terpineol, an amphipathic monoterpene alcohol, is known to compromise parasite membrane integrity and may trigger apoptosis-like mechanisms^46^. Pomegranate extracts also contain aromatic hydrocarbons such as *p*-cymene and *m*-cymene. While these compounds may exhibit limited standalone antiparasitic activity, their ability to enhance membrane fluidity and permeability can facilitate the uptake of more potent monoterpenes, thereby functioning as synergistic enhancers of overall extract efficacy. Additionally, fatty acid derivatives, including palmitic acid and monopalmitin, represent another key class of compounds. Saturated fatty acids like palmitic acid are known to influence membrane dynamics, potentially disrupting essential parasite functions. Monopalmitin, a monoester of palmitic acid, exhibits antimicrobial and antiparasitic effects, possibly by targeting surface lipids or disrupting biofilm formation^47,48^.

This study aimed to evaluate the antiparasitic efficacy of *Punica granatum* (pomegranate) extract against *Caligus clemensi*, with a particular focus on the molecular interactions between the plant-derived compounds and crustacean proteins. A novel feature of this research is the integration of in silico molecular docking analyses to elucidate the binding affinities and potential inhibitory mechanisms of *P. granatum* constituents on key *C. clemensi* proteins. This combined computational and experimental approach advances the understanding of the extract’s parasiticidal mode of action, offering valuable insights for the development of plant-based therapeutics in sustainable aquaculture.

## Materials and methods

### Ethical approval

This study’s methods and trial protocols complied with the relevant guidelines and regulations for the use of fish in research, as stipulated by the American Fisheries Society^49^. Approval was granted by the institutional Animal Care and Use Committee Beni-Suef University (BSU-IACUC 025-022)

### Collection of crustacean parasites

This study was conducted in the Deeba Triangle region (31° 24′ 46″ N, 31° 52′ 38″ E), a significant aquaculture zone located in the northern Nile Delta of Egypt. The Deeba Triangle is bordered to the north by the Mediterranean Sea, to the west by the Damietta Branch of the Nile River, and to the south by Lake Manzala, creating a unique triangular aquaculture ecosystem. The investigation was initiated in response to significant mortality observed among farmed European seabass (*Dicentrarchus labrax*) and gray mullet (*M. cephalus*) at a private aquaculture facility (El-Deepa Farm) located within this area.

A total of 800 diseased fish samples—400 from European sea bass (*D. labrax*) and 400 from flathead gray mullet (*M. cephalus*)—were randomly taken from the outbreak farm in the Deeba region. Using isothermal containers loaded with crushed ice, the obtained samples were quickly moved to the laboratory.

Following necropsy, the skin and gills of each fish were scraped off and examined under a dissecting microscope. Copepods were manually isolated from skin and gill surfaces under stereomicroscope examination for subsequent molecular and morphological analyses.

### Small subunit rRNA gene sequencing and phylogenetic analysis

Total genomic DNA was isolated from six representative copepod specimens (three from European seabass and three from flathead gray mullet) using the DNeasy Blood and Tissue Kit (Qiagen, Valencia, CA, USA). DNA purity and concentration were assessed via a NanoDrop spectrophotometer (ACT-Gene, Thermo Fisher Scientific, Waltham, MA, USA). The small ribosomal subunit rRNA gene was amplified for taxonomic identification via primer sets from Abdelkhalek et al.^50^. Amplification reactions were conducted via MyTaq Red Mix (Bioline, Taunton, MA, USA) in a total volume of 50 µl, consisting of 25 µl of MyTaq Red Mix, 2 µl of each primer (10 µM), 5 µl of template DNA, and 16 µl of sterile distilled water. PCR was performed under the following conditions: initial denaturation at 95 °C for 10 min, followed by 35 amplification cycles (denaturation at 95 °C for 30 s, primer annealing at 50 °C for 30 s, extension at 72 °C for 60 s), and a final extension step at 72 °C for 7 min. The PCR products were examined on agarose gels, extracted via the QIAquick Gel Extraction Kit (QIAGEN, Hilden, Germany), and subjected to bidirectional sequencing at Macrogen, Inc. (Seoul, Korea). The raw sequences were processed and edited via BioEdit software^51^ and then submitted to GenBank following BLAST analysis against existing Caligidae reference sequences. All six sequences were deposited in GenBank. Sequencing was performed on all six specimens, with sequences showing > 98% similarity confirming identification as *C. clemensi*. Multiple sequence alignment and phylogenetic reconstruction were conducted via the maximum likelihood method in MEGA11^52^. The GTR + G + I substitution model was selected on the basis of optimal model selection criteria. *Sapphirina nigromaculata* (LC711103) served as the outgroup taxon for tree rooting.

### Electron microscopy analysis

Scanning electron microscopy was employed to analyze the ultramorphological features of the *Caligid* copepod parasites. The parasites were preserved in a 2.5% glutaraldehyde solution and dehydrated through a sequential series of ethanol concentrations (50%, 70%, 90%, and 100%) for 10 min per concentration. The whole parasitic copepod sample was dehydrated via a CO_2_ critical point drier (Autosamdri-815, Germany). The copepods were mounted onto scanning electron microscope stubs, coated with a 20 nm layer of gold via a sputter coater (Spi-Module sputter coater, UK), and imaged via a JEOL JSM 5200 electron probe microanalyzer at the Faculty of Agriculture, Cairo University, Egypt. All the measurements are expressed in millimeters.

### Preparation of plant extract

Fresh pomegranate (*P. granatum*) fruits were obtained from local markets in Cairo, Egypt, and authenticated by the Botany Department, Faculty of Science, and Cairo University. The peels were carefully separated from the fruits, washed thoroughly with distilled water to remove any debris, and shade-dried at room temperature (25 ± 2 °C) for 7 days until completely dry. The dried peels were ground into a fine powder using an electric grinder and passed through a 40-mesh sieve to ensure uniform particle size.

For methanolic extraction, 100 g of pomegranate peel powder was macerated in 500 mL of 80% methanol (v/v) at room temperature with continuous stirring for 72 h. The mixture was filtered through Whatman No. 1 filter paper, and the filtrate was concentrated using a rotary evaporator (Buchi R-210, Switzerland) at 40 °C under reduced pressure. The dried extract was weighed, and the extraction yield was calculated. For bioassays, the dried extract was dissolved in dimethyl sulfoxide (DMSO) to prepare a stock solution of 100 mg/mL, and stored at 4 °C until use. The final yield of the extract was calculated as a percentage of the initial dry weight.

### Gas chromatography-mass spectrometry (GC-MS) analysis

The chemical composition of the pomegranate methanolic extract was analyzed using GC-MS. Prior to analysis, the extract was dissolved in HPLC-grade methanol (1 mg/mL) and filtered through 0.22 μm syringe filters. The analysis was performed in triplicate using a Thermo Scientific Trace GC Ultra coupled with a Thermo Scientific ISQ Single Quadrupole MS (Thermo Fisher Scientific, USA). The system was equipped with a TG-5MS fused silica capillary column (30 m × 0.25 mm i.d., 0.25 μm film thickness).

The oven temperature was programmed from 60 °C (held for 2 min) to 280 °C at a rate of 5 °C/min, with a final hold time of 10 min. Helium was used as the carrier gas at a constant flow rate of 1.0 mL/min. The injection volume was 1 µL with a split ratio of 1:50. The injector and MS transfer line temperatures were set at 250 °C and 280 °C, respectively.

The mass spectrometer was operated in electron impact (EI) mode at 70 eV. The ion source temperature was 200 °C, and the mass scan range was 50–750 m/z. Compounds were identified by comparing their retention times and mass spectra with those in the NIST Mass Spectral Library (NIST 14) and Wiley Registry of Mass Spectral Data. Peak identification was confirmed with a minimum match factor of ≥ 80%. The relative percentage of each component was calculated by comparing its peak area to the total peak area.

### In vitro study of the tested plant extracts

The toxicity of pomegranate (*P. granatum*) methanolic extract against *C. clemensi* adults was evaluated by using a range of concentrations (0.63%, 1.25%, 2.5%, 5%, and 10%) prepared in dechlorinated tap water containing 0.05% Tween 80 as an emulsifying agent. The extract concentrations were prepared from the stock solution (100 mg/mL in DMSO) by serial dilution. Each concentration was tested on 50 crustacean individuals, divided into five replicates (10 individuals per replicate), each housed in a 100 mL Petri dish. Control groups were exposed to dechlorinated tap water with 0.05% Tween 80 and the equivalent DMSO concentration without extract. Lethal concentrations (LC₅₀ and LC₁₀₀) were determined based on observed mortality at various time points (15 min, 30 min, 1 h, 2 h, 4 h, 8 h, 12 h, 24 h, and 48 h).

### In silico methodology

#### Protein structure retrieval

**Fig. 1:**
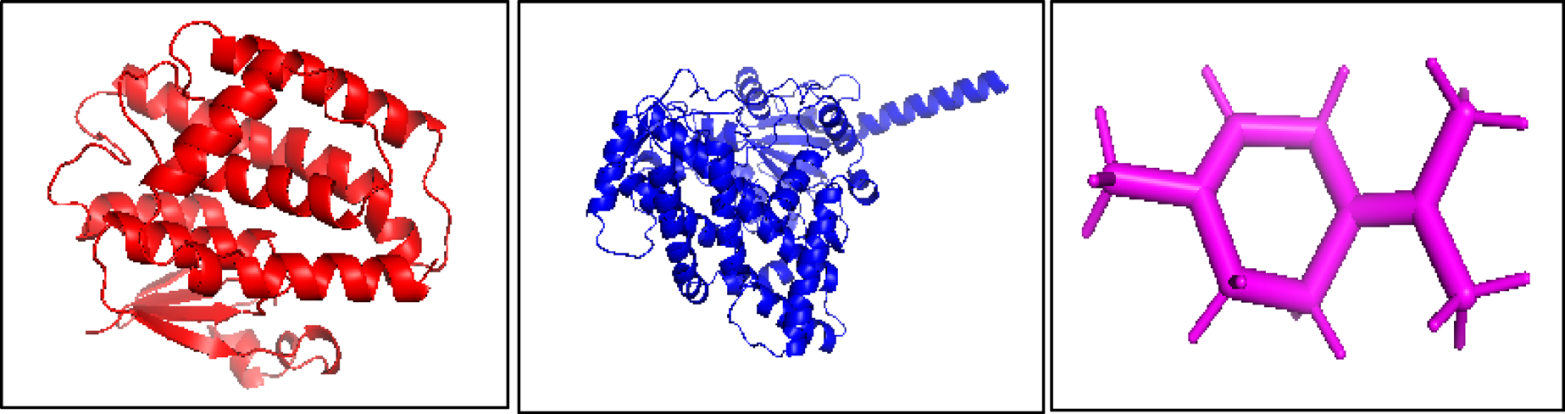
Molecular structure of protein representations of Glutathione S-transferase theta 1 (GSTT1) and Cytochrome P450 3A24 and cyclohexene: (**a**) Glutathione S-transferase theta 1 (GSTT1)—a detoxification enzyme facilitating the conjugation of glutathione with toxic compounds, essential for cellular protection. (**b**) Cytochrome P450 3A24—a key enzyme in xenobiotic and drug metabolism, playing a crucial role in biotransformation and detoxification pathways. (**c**) Cyclohexene—a six-membered cyclic alkene featuring a double bond, influencing its chemical reactivity and interactions.

Three-dimensional (3D) structures of glutathione S-transferase theta 1–1 (GSTT1) and cytochrome P450 3A24, both from *C. clemensi*, were retrieved from the UniProtKB/TrEMBL database (UniProt IDs: C1C2N8 and C1C148, respectively). GSTT1 is a phase II detoxification enzyme that catalyzes the conjugation of glutathione to xenobiotic compounds, playing a crucial role in parasite resistance to toxins. Cytochrome P450 3A24 is a phase I metabolic enzyme involved in the oxidative metabolism of endogenous and exogenous compounds, essential for parasite survival and drug resistance. Structural data were obtained in Protein Data Bank (PDB) format and visualized via UCSF Chimera for further preparation and analysis (Fig. 1a and b).

#### Ligand preparation

The chemical compound 1-methyl-4-(1-methylethylidene)cyclohexene (C₁₀H₁₆; MW: 136 g/mol; CAS No. 586-62-9) was selected as the ligand based on its abundance in the GC-MS analysis (5.27% of total extract composition) and its structural properties suitable for molecular docking studies. This monoterpene was chosen over more complex polyphenols or tannins due to its well-defined structure and documented bioactivity. Its 2D structure was sourced from the ZINC database (ZINC ID: 23371). The SMILES canonical representation (CC(C) = C1CC = C(C)CC1) was used to generate a 3D conformer via UCSF Chimera. Geometry optimization was performed to minimize steric strain and obtain a stable structure (Fig. 1c).

#### Protein preparation for docking

The retrieved protein structures were preprocessed using UCSF Chimera to ensure suitability for molecular docking simulations. This preprocessing involved the removal of nonstandard residues and crystallographic water molecules, addition of polar hydrogen atoms, and assignment of Gasteiger charges. Subsequently, energy minimization was performed using default parameters to optimize protein geometry and relieve steric clashes. These steps collectively prepared the protein structures for accurate docking analyses.

#### Active site prediction

The active binding pockets within GSTT1 and Cytochrome P450 3A24 were identified via the SCFBio Active Site Prediction server (http://www.scfbio-iitd.res.in/dock/ActiveSite.jsp). The server provided coordinates for putative ligand-binding regions on the basis of geometric and electrostatic criteria. These regions guided the definition of grid parameters for docking.

#### Molecular docking

Molecular docking was carried out via AutoDock Vina integrated into UCSF Chimera^53^. The reliability of molecular docking predictions for natural products has been extensively evaluated, with emphasis on proper validation procedures^54^. Grid box dimensions were defined around the predicted active sites for both proteins. The docking parameters included an exhaustiveness of 8, with multiple runs performed to ensure reproducibility. The top-ranked binding poses were selected on the basis of binding affinity (kcal/mol) and orientation within the active site.

#### Post-docking interaction analysis

Protein‒ligand interaction profiles were generated via the Protein‒Ligand Interaction Profiler (PLIP) server (https://plip-tool.biotec.tu-dresden.de). PLIP provides detailed reports of noncovalent interactions, including hydrogen bonds, hydrophobic contacts, π‒π stacking, and salt bridges. These interactions were used to evaluate binding specificity and potential inhibitory mechanisms.

#### Molecular dynamics and flexibility analysis

To assess protein‒ligand complex stability and flexibility, normal mode analysis (NMA) was performed via the iMODS server (https://imods.iqf.csic.es/). The analysis generated eigenvalues, deformability plots, and B-factor estimations, providing insights into the dynamic behavior of the complexes under simulated physiological conditions.

### Statistical analysis

Statistical analysis of the data was performed via the Statistical Package for Social Science (SPSS for Windows (IBM), version 22, Chicago, USA). ANOVA tests and subsequent Duncan’s multiple range tests were applied to determine the differences between means. The data are presented as the means, and the values were considered significant at *P* < 0.05. The effective concentration (LC50) with a 95% confidence interval (LC 100%) was calculated (SPSS version 22).

## Results

### Molecular phylogenetic characterization of parasitic copepods

Six representative samples of parasitic copepods were molecularly characterized, including three specimens from European seabass (*D. labrax*) and three from flathead gray mullet (*M. cephalus*). A 1,020 bp fragment of the 18 S rRNA gene was amplified and sequenced from parasitic copepod samples. BLAST analysis confirmed taxonomic placement as *C. clemensi*, with all six sequences showing > 99% similarity to reference *C. clemensi* isolates and being identical to each other. The sequences were deposited in GenBank under accession OR563780 and PV955728-PV955732. Pairwise comparisons revealed 99.71–99.90% identity with conspecific *C. clemensi* isolates (PP837597, MT151385, and DQ123833), confirming species identification. Sequence similarity decreased systematically with phylogenetic distance: congeneric *Caligus* species (96.68–97.95%, closest to *C. elongatus* at 97.95%), confamilial *Lepeophtheirus* species (95.19–96.39%), other parasitic copepods (90.39–91.72%), and the outgroup *S. nigromaculata* (87.39%). Evolutionary distance analysis via the GTR + G + I substitution model corroborated these relationships, with pairwise distances ranging from 0.001 (conspecific) to 0.147 (outgroup), demonstrating clear taxonomic separation across all levels (Table 1).

**Table 1:** Pairwise evolutionary distances (lower triangle) and percentage similarity values (upper triangle) among *Caligus clemensi* and related copepod species based on 18 S rRNA sequences.

Species	C.clem1 (OR563780)	C.clem2 (MT151385)	C.elon (EF088409)	C.curt (MF077737)	C.lala (MW925123)	C.roge (AY174144)	L.salm (AF208263)	L.poll (EF088414)	L.bran (MN523344)	Pen.sp (LC586438)	S.nigr (LC711103)
*C. clemensi* (OR563780)	–	99.9	98	97.6	97.3	98.2	96.2	96.2	91.2	89.9	85.3
*C. clemensi* (MT151385)	0.001	–	97.9	97.5	97.2	98.2	96.1	96.1	91.1	89.8	85.2
*C. elongatus* (EF088409)	0.02	0.021	–	98.1	97.4	98.4	96.5	96.4	91.5	90.2	85.6
*C. curtus* (MF077737)	0.024	0.025	0.019	–	97.1	98	97.2	97	91.9	90.6	86
*C. lalandei* (MW925123)	0.027	0.028	0.026	0.029	–	97.7	95.6	95.5	90.7	89.4	84.8
*C. rogercresseyi* (AY174144)	0.018	0.018	0.016	0.02	0.023	–	96.6	96.5	92.1	90.8	86.7
*L. salmonis* (AF208263)	0.038	0.039	0.035	0.028	0.044	0.034	–	99.6	92.2	90.9	86.3
*L. pollachius* (EF088414)	0.038	0.039	0.036	0.03	0.045	0.035	0.004	-	92.1	90.8	86.2
*L. branchialis* (MN523344)	0.088	0.089	0.085	0.081	0.093	0.079	0.078	0.079	-	98.8	84.5
*Pennella* sp. (LC586438)	0.101	0.102	0.098	0.094	0.106	0.092	0.091	0.092	0.012	-	83.2
*S. nigromaculata* (LC711103)	0.147	0.148	0.144	0.14	0.152	0.133	0.137	0.138	0.155	0.168	-

Estimated pairwise evolutionary distances based on 18 S rRNA gene sequences for *C. clemensi*. Maximum likelihood estimation with complete deletion of gaps. Number of nucleotide sites in final analysis: 1020 bp. Analysis conducted in MEGA11 with 1000 bootstrap replicates. Analysis conducted in MEGA11 with 1000 bootstrap replicates. Distance values represent nucleotide substitutions per site; similarity values represent percentage identity. Species abbreviations: *C.* = *Caligus*; *L.* = *Lepeophtheirus*; *L.* = *Lernaocera*; *Pen.* = *Pennella*; *S.* = *Sapphirina.*

**Fig. 2:**
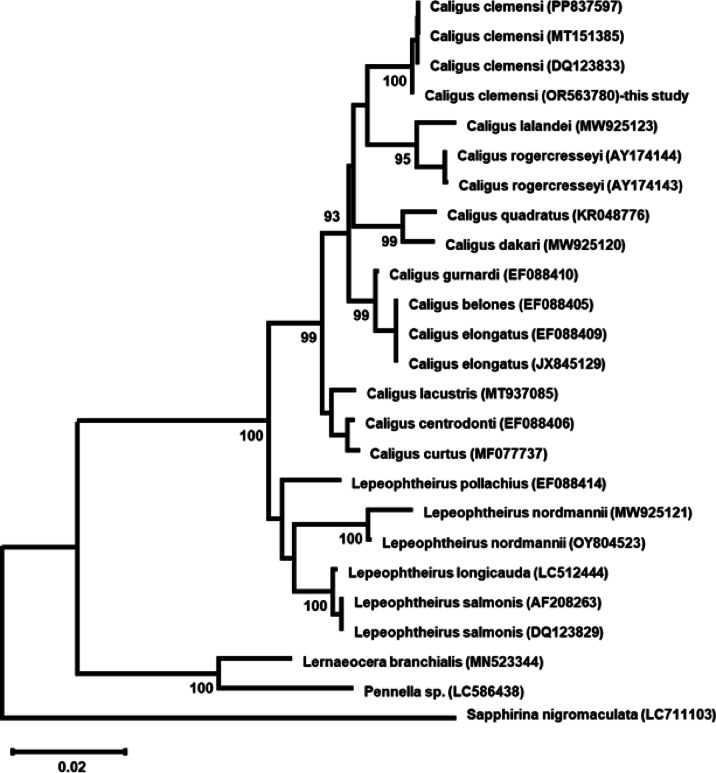
Maximum likelihood phylogenetic tree of *C. clemensi* (OR563780) from this study based on 18S rRNA gene sequences. *S. nigromaculata* (LC711103) served as the outgroup. The scale bar represents 0.02 nucleotide substitutions per site. The phylogeny demonstrates clear generic separation between *Caligus* and *Lepeophtheirus* within Caligidae, while *Lernaocera* and *Pennella* represent distinct parasitic copepod lineages. All major clades received strong bootstrap support, validating the robustness of the phylogenetic relationships inferred.

Maximum likelihood reconstruction produced a well-resolved tree with two main Caligidae clades (Fig. 2). Our *C. clemensi* specimens formed a monophyletic group with other conspecific isolates (100% bootstrap), which clustered closely with *C. lalandei* and *C. rogercresseyi* (95% bootstrap). The *Caligus* clade was subdivided into several well-supported subclades (≥ 93% bootstrap), whereas the *Lepeophtheirus* species formed a distinct sister clade with a clear internal structure. *Lernaocera branchialis* and *Pennella* sp. represented separate parasitic lineages (100% bootstrap each), with *S. nigromaculata* serving as an appropriate outgroup.

### GC-MS analysis of pomegranate methanolic extract

Gas chromatography-mass spectrometry analysis of the pomegranate methanolic peel extract revealed a complex chemical profile with 28 distinct compounds identified (Table 2; Supplementary File 1). The major constituents included monoterpenes (45.2%), aromatic hydrocarbons (24.1%), and fatty acid derivatives (30.7%). Among the monoterpenes, 1-methyl-4-(1-methylethylidene) cyclohexene was the most abundant (5.27%), followed by γ-terpinene (15.03%), while terpineol (34.46%) represented the major oxygenated monoterpene. The aromatic hydrocarbon fraction was dominated by p-cymene (6.54%) and m-cymene. Fatty acid derivatives included palmitic acid (21.13%) and its trimethylsilyl ester (17.57%). The selection of 1-methyl-4-(1-methylethylidene)cyclohexene for molecular docking analysis was based on several criteria: (1) it represents a structurally well-defined monoterpene amenable to computational modeling, (2) similar compounds have demonstrated antiparasitic activity in previous studies, and (3) its moderate abundance (5.27%) and chemical structure make it a representative compound for studying monoterpene-protein interactions.

**Table 2:** Gas chromatography-mass spectrometry (GC-MS) analysis of *Punica granatum* methanolic Peel extract showing identified compounds, their retention times, molecular characteristics, and relative abundance.

RT (min)	Compound name	Molecular formula	MW (g/mol)	CAS number	Area (%)	Match factor	Compound class
5.04	1-Methyl-4-(1-methylethylidene)cyclohexene	C₁₀H₁₆	136	586-62-9	5.27	899	Monoterpene
5.1	p-Cymene	C₁₀H₁₄	134	99-87-6	6.54	842	Aromatic hydrocarbon
5.86	γ-Terpinene	C₁₀H₁₆	136	99-85-4	15.03	938	Monoterpene
8.46	3-Cyclohexen-1-ol, 4-methyl-1-(1-methylethyl)- (Terpineol)	C₁₀H₁₈O	154	562-74-3	34.47	925	Monoterpene alcohol
26.53	Hexadecanoic acid (Palmitic acid)	C₁₆H₃₂O₂	256	57-10-3	21.13	864	Fatty acid
28.23	Palmitic acid, TMS derivative	C₁₉H₄₀O₂Si	328	520-89-3	17.57	792	Fatty acid derivative

Total identified compounds: 99.00% RT: Retention time; MW: Molecular weight; CAS: Chemical Abstracts Service registry number; TMS: Trimethylsilyl. Major compound classes identified: -Monoterpenes and derivatives: 61.30% (α-terpinene complex, γ-terpinene, terpineol) -Aromatic hydrocarbons: 6.54% (p-cymene) -Fatty acids and derivatives: 38.70% (palmitic acid and TMS derivative).

Compound identification was based on comparison with NIST mass spectral library with match factors > 800.

The monoterpene 1-methyl-4-(1-methylethylidene)cyclohexene was selected for molecular docking studies based on its structural simplicity, known antiparasitic properties of monoterpenes, and suitability for computational modeling.

### In vitro efficacy of pomegranate methanolic extract activity against Caligus sp.

**Fig. 3:**
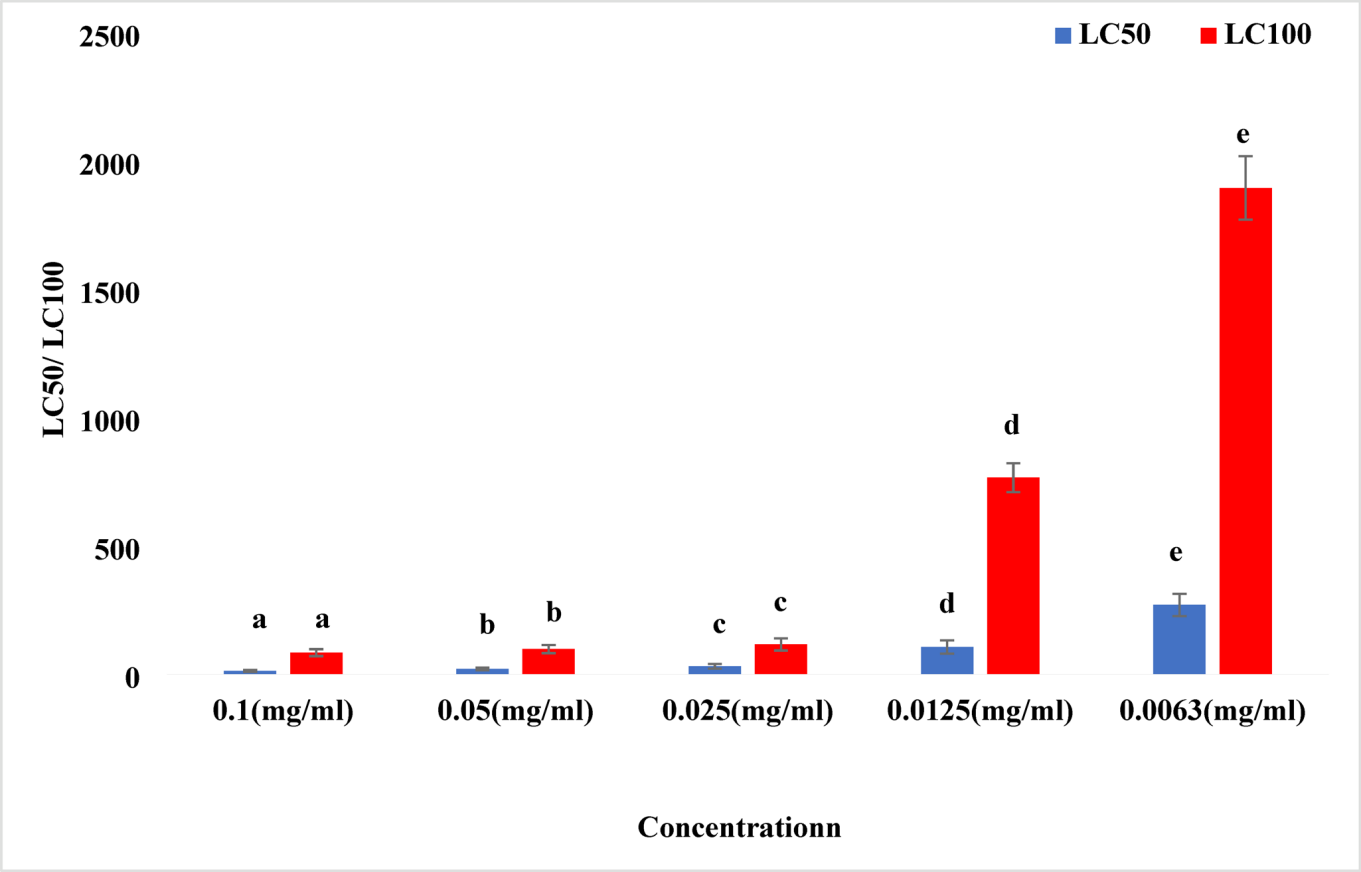
Lethal concentrations (LC50 and LC100) of *Punica granatum* methanolic extract against *Caligus clemensi* at different exposure concentrations. Blue bars represent LC50 values, and red bars represent LC100 values. Different letters (a–e) above bars indicate statistically significant differences between treatment groups according to Duncan’s multiple range test (*p* < 0.05). Bars with the same letter are not significantly different. The dose-dependent relationship demonstrates increased toxicity with higher extract concentrations.

**Fig. 4:**
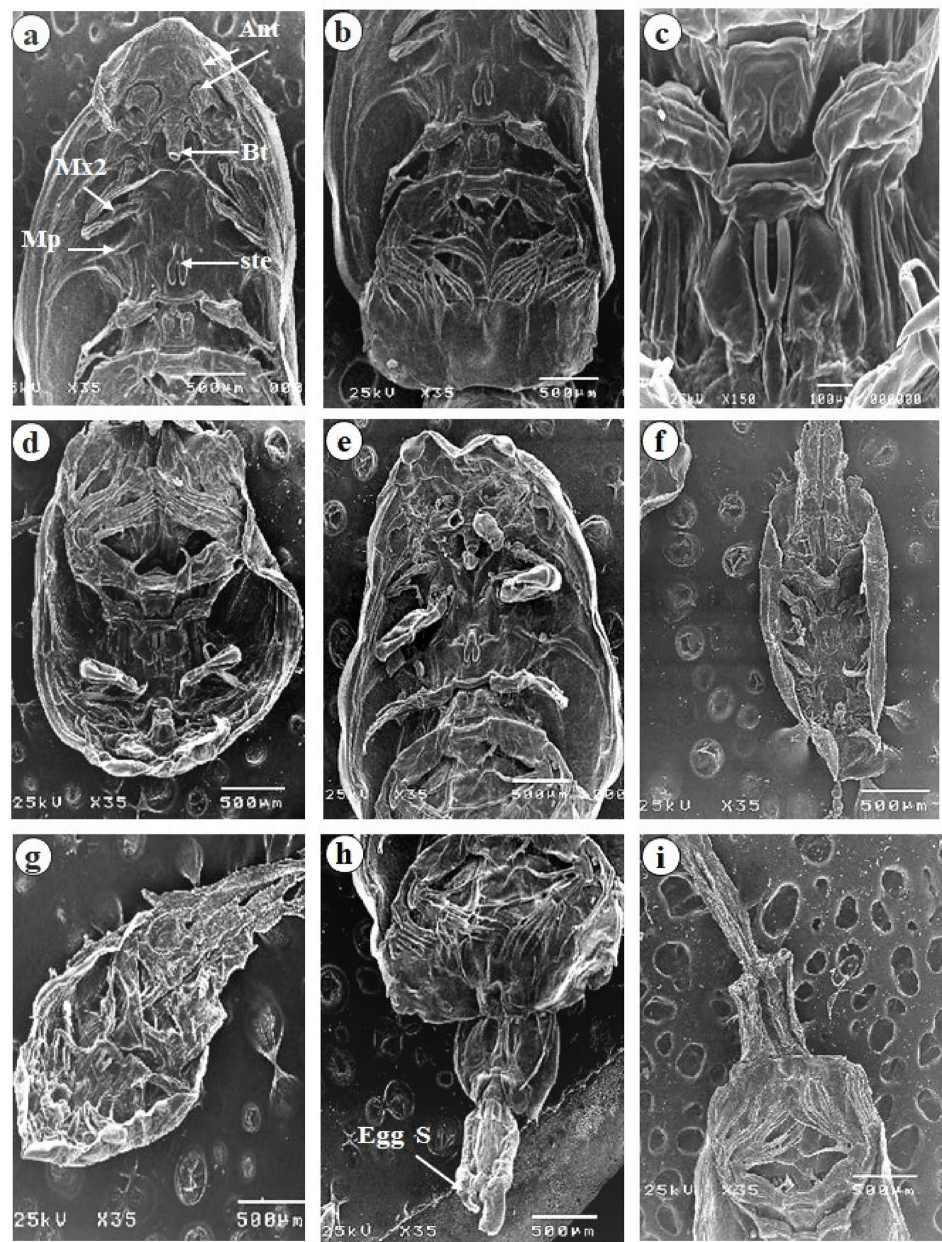
Scanning electron microscope study of *C. clemensi*, showing normal control cuticle in (**a–c**), including Ant (antenna), BT (buccal tube), Egg S (egg sacs), Ste (sternal furca), Mp (maxillipeds), and Mx2 (maxilla). (**d–i**) display a variable degree of shrinkage in the cuticle and egg sacs, highlighting structural alterations.

Lethal concentrations and their corresponding exposure times for different treatments of pomegranate (*Punica granatum*) methanolic extract activity against *Caligus* sp. were assessed. At a concentration of 10%, the LC50 was reached after 12.37 min. Moreover, at a concentration of 5%, the LC50 was reached at 20.28 min. For concentrations of 2.5, 1.25 and 0.63%, the LC50 values were 30.63 min, 1.76 h and 4.49 h, respectively. These values suggest that increasing the concentration of the methanolic extract significantly decreases the time required to achieve 50% mortality of *Caligus* sp. The LC100, which represents the concentration required to kill 100% of the examined *Caligus* sp., was 1.39 h, 1.62 h, 1.93 h, 12.75 h and 31.57 h at concentrations of 10, 5, 2.5, 1.25 and 0.63%, respectively; Table 3; Fig. 3. Scanning microscopic analysis of the treated crustacean parasites revealed various degrees of shrinkage in different areas, such as the cephalothorax and abdomen and egg sacs (Fig. 4).

**Table 3:** Lethal concentrations (LC₅₀ and LC₁₀₀) of pomegranate (*Punica granatum*) methanolic extract against *Caligus* sp. and their associated exposure times.

Concentration %	LC_50_	LC_100_
10%	12.37 min	1.39 h
5%	20.28 min	1.62 h
2.5%	30.63 min	1.93 h
1.25%	1.76 h	12.75 h
0. 63%	4.49 h	31.57 h

Values represent mean exposure times required to achieve 50% (LC₅₀) and 100% (LC₁₀₀) mortality. Different concentrations showed statistically significant differences (*p* ≤ 0.05).

### In silico study

The active sites of glutathione S-transferase theta 1–1 (GSTT1) and cytochrome P450 3A24 were successfully predicted via cavity detection methods. For GSTT1, the predicted binding pocket, labeled cavity_1_YPMVASNQERFHGKI, was located at the coordinates x = − 4.118, y = − 5.835, and z = − 9.403. Similarly, cytochrome P450 3A24 exhibited a prominent cavity, cavity_1_KQTFIYLSEPMARNCDHGVW, centered at x = 5.690, y = − 12.349, and z = 7.582. A uniform docking grid box of 20 × 20 × 20 Å³ was applied to both targets to encompass their entire active sites for subsequent docking procedures. The accuracy of the docking poses was confirmed by a root mean square deviation (RMSD) of 0 Å in each case, indicating highly consistent and reproducible docking results. The docking affinity of the ligand, 1-methyl-4-(1-methylylidene) cyclohexene, at position 1 (POS1) was − 4.9 kcal/mol for GSTT1 and − 5.0 kcal/mol for cytochrome P450 3A24, reflecting favorable binding interactions with both protein targets.

**Fig. 5:**
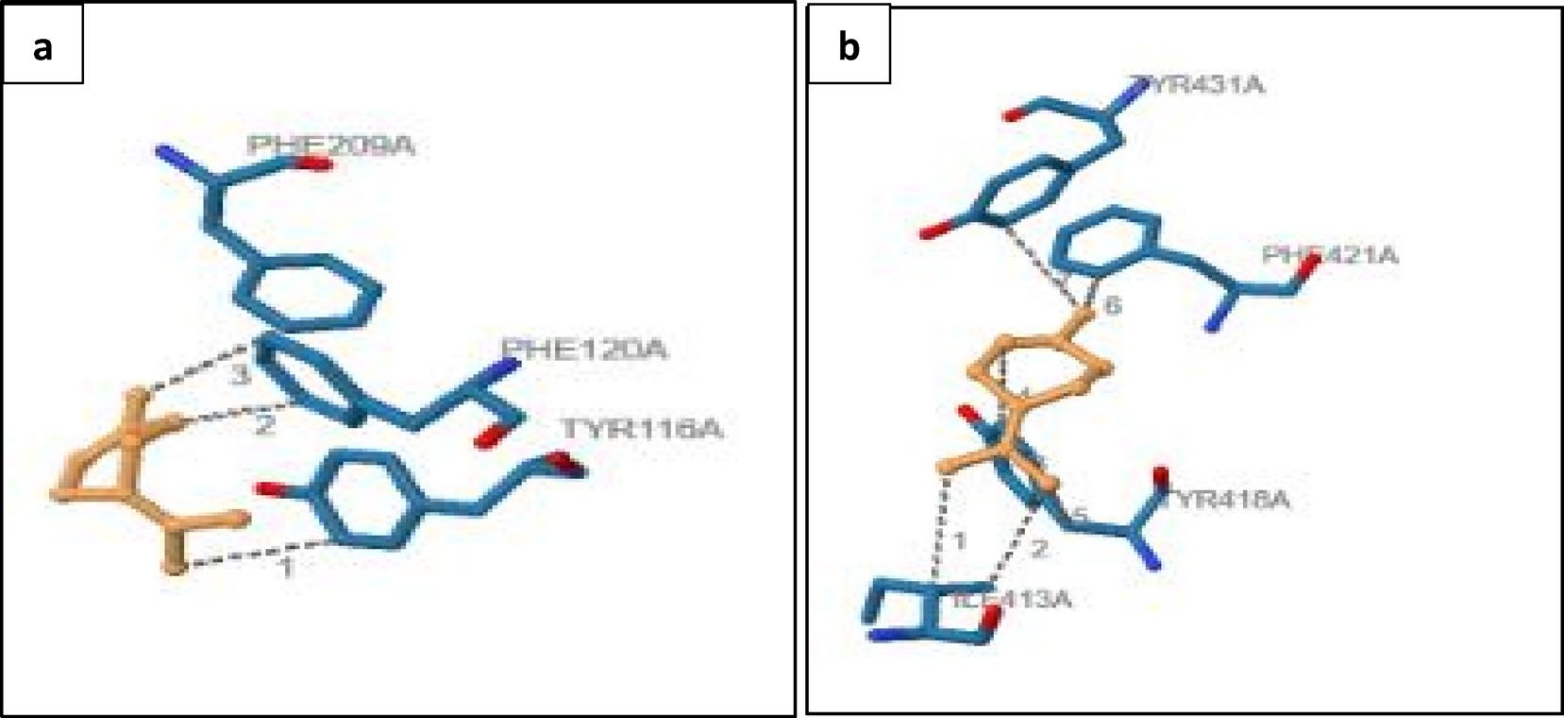
Interaction cyclohexene with detoxification enzymes. **(a)** The docked complex of Glutathione S-transferase theta 1 (GSTT1) with Cyclohexene. The figure illustrates the binding conformation of Cyclohexene within the active site of GSTT1, highlighting key interactions such as hydrophobic contacts and potential hydrogen bonds. The ligand is shown in stick representation, while the protein surface or cartoon representation demonstrates the spatial accommodation of the molecule. This visualization provides insights into the ligand orientation and possible metabolic interactions mediated by GSTT1. **(b)** The docked complex of Cytochrome P450 3A24 with Cyclohexene. The binding pose of the ligand is displayed within the enzyme’s active site cavity, with specific emphasis on proximity to the heme group (if shown), suggesting its potential oxidative metabolism. Residue-ligand interactions are visualized to assess binding affinity and compatibility, offering clues about the role of CYP3A24 in processing Cyclohexene through monooxygenation or other catalytic reactions.

To elucidate the nature of these interactions, the protein–ligand interaction profiler (PLIP) was used to analyze the complexes. In GSTT1, the ligand was stabilized primarily through hydrophobic interactions involving residues TYR116A, PHE120A, and PHE209A (Fig. 5a; Table 4). These interactions suggest that the ligand fits snugly within the hydrophobic pocket of the protein, enhancing binding stability. For Cytochrome P450 3A24, PLIP analysis revealed multiple hydrophobic contacts, notably with residues ILE413A (two interactions), TYR418A (three interactions), PHE421A (one interaction), and TYR431A (one interaction) (Fig. 5b; Table 5). TYR418A, which contributes three hydrophobic contacts, appears to play a dominant role in anchoring the ligand, likely through π‒π stacking or van der Waals interactions mediated by its aromatic ring. The dual contacts formed by ILE413A further support strong binding, whereas the contributions from PHE421A and TYR431A reinforce ligand positioning within the enzyme’s active site.

**Table 4:** PLIP result (GSTT1-cyclohexene) complex.

Hydrophobic Hydrophobic					
Index	Residue	AA	Distance	Ligand atom	Protein atom
1	116 A	TYR	3.61	1718	914
2	120 A	PHE	6.60	1726	950
3	209 A	PHE	3.73	1725	1649

The table presents the PLIP (Protein-Ligand Interaction Profiler) results for the GSTT1-Cyclohexene complex, detailing key molecular interactions. It highlights binding residues, interaction types, such as hydrogen bonds, hydrophobic interactions, and π-stacking, along with their respective distances and strengths. This analysis provides insights into the stability and binding affinity of Cyclohexene within the GSTT1 active site.

**Table 5:** PLIP results (cytochrome P450 3A24- cyclohexene) complex.

Hydrophobic interactions					
Index	Residue	AA	Distance	Ligand atom	Protein atom
1	413 A	ILE	3.86	3913	3352
2	413 A	ILE	3.65	3915	3355
3	418 A	TYR	3.52	3914	3393
4	418 A	TYR	3.76	3918	3395
5	418 A	TYR	3.76	3915	3390
6	421 A	PHE	3.62	3920	3424
7	431 A	TYR	3.69	3920	3499

This table presents the PLIP (Protein-Ligand Interaction Profiler) results for the Cytochrome P450 3A24-Cyclohexene complex, detailing molecular interactions between Cyclohexene and active site residues of Cytochrome P450 3A24. It highlights binding residues, interaction types such as hydrogen bonds, hydrophobic contacts, π-stacking, and salt bridges, along with their respective distances and strengths. These results provide insights into binding stability, affinity, and the molecular recognition mechanisms within the active site.

These findings indicate a dual inhibitory mechanism potentially underlying the antiparasitic activity of 1-methyl-4-(1-methylylidene)cyclohexene derived from *Punica granatum*. First, interaction with GSTT1 may inhibit detoxification processes within *C. clemensi* by occupying hydrophobic binding regions, thus interfering with glutathione conjugation and reducing parasite survival under stress. Second, the more extensive and complex interaction profile observed for Cytochrome P450 3A24 suggests that this ligand may disrupt essential metabolic pathways by obstructing the enzymatic activity central to parasite development. The greater number of stabilizing interactions in P450 also implies a stronger inhibitory effect, possibly hindering redox reactions or substrate turnover, which are critical for parasite viability. By targeting both detoxification and metabolic enzymes, this compound may reduce the likelihood of resistance development, especially when combined with host immune responses. Overall, these mechanistic insights highlight the promise of pomegranate-derived cyclohexene as a sustainable and multitarget antiparasitic agent, although further validation using parasite-specific proteins and in vivo studies is essential.

**Fig. 6:**
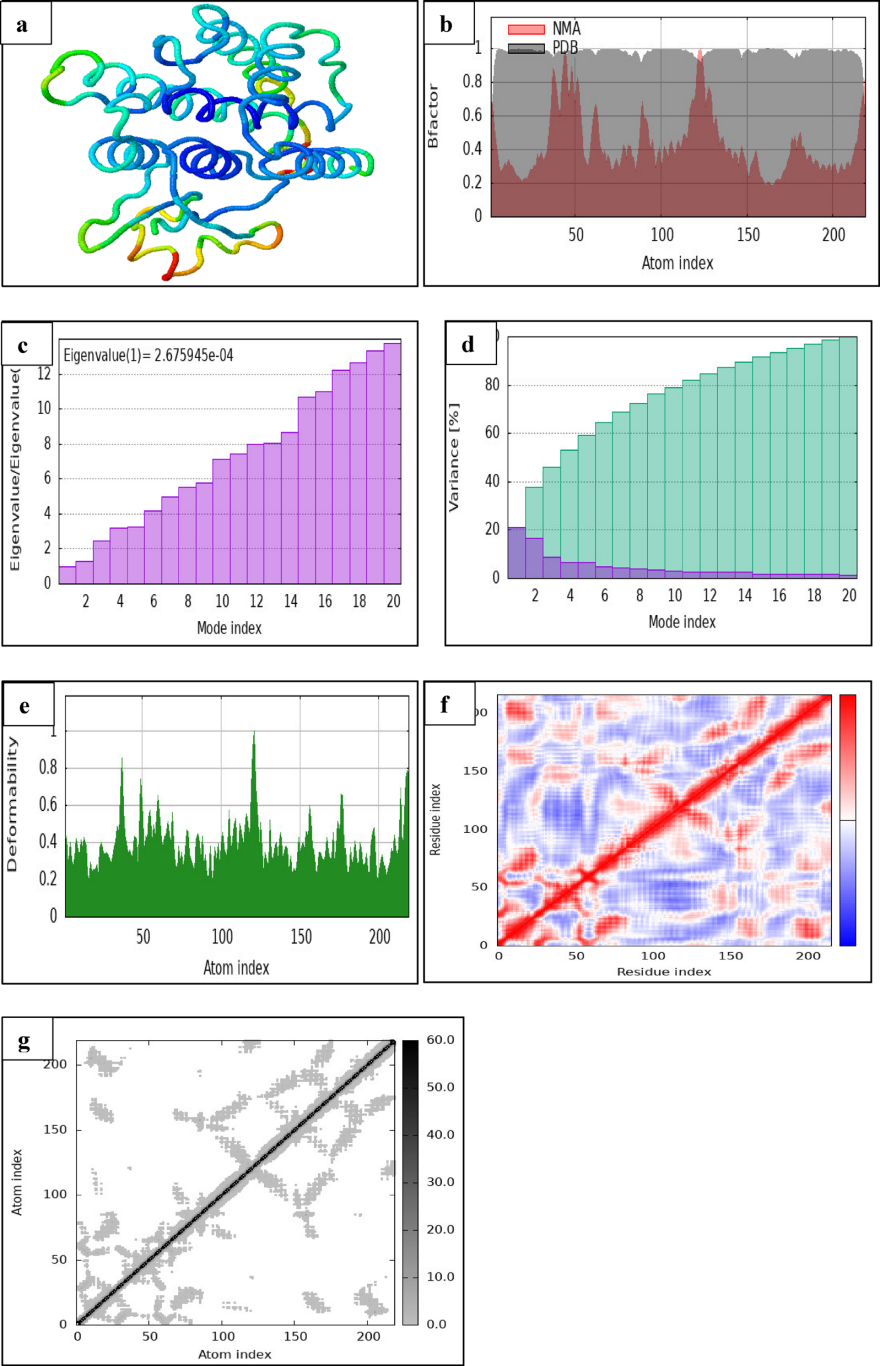
iMODS Results for the Interaction of 1-Methyl-4-(1-Methylethylidene)cyclohexene with Glutathione S-transferase theta 1 (GSTT1). (**a**) 3D protein structure displaying secondary structural elements in blue, with a green-to-red gradient highlighting a functionally significant region, potentially involved in binding or structural flexibility. (**b**) B-factor plot comparing atomic flexibility between Normal Mode Analysis (NMA) and Protein Data Bank (PDB) data. The PDB dataset exhibits higher B-factors, indicating greater atomic movement in experimental conditions. (**c**) Eigenvalue graph illustrating the relationship between mode index and eigenvalue ratios, showing a steady increase, which is essential for dimensionality reduction and vibrational analysis. A key annotation “Eigenvalue (1) = 2.675945e-04” serves as a reference point. (**d**) Variance plot showing individual and cumulative variance distributions across mode indices. Purple bars represent higher individual variance contributions, while green bars indicate cumulative accumulation, helping identify dominant structural modes. (**e**) Deformability plot, mapping atomic flexibility variations. Higher peaks correspond to flexible regions, while troughs indicate rigid segments, aiding in the interpretation of molecular dynamics and behavior. (**f**) Co-variance map, residue-residue interaction heatmap, depicting protein connectivity through contact intensity. Red zones mark strong interactions, while off-diagonal elements highlight key folding and binding sites. (**g**) Elasticity map, Atomic interaction heatmap, visualizing interatomic correlations. Color intensity reflects interaction strength, with diagonal trends showing self-correlation and off-diagonal clusters indicating molecular stability and dynamic behavior.

**Fig. 7:**
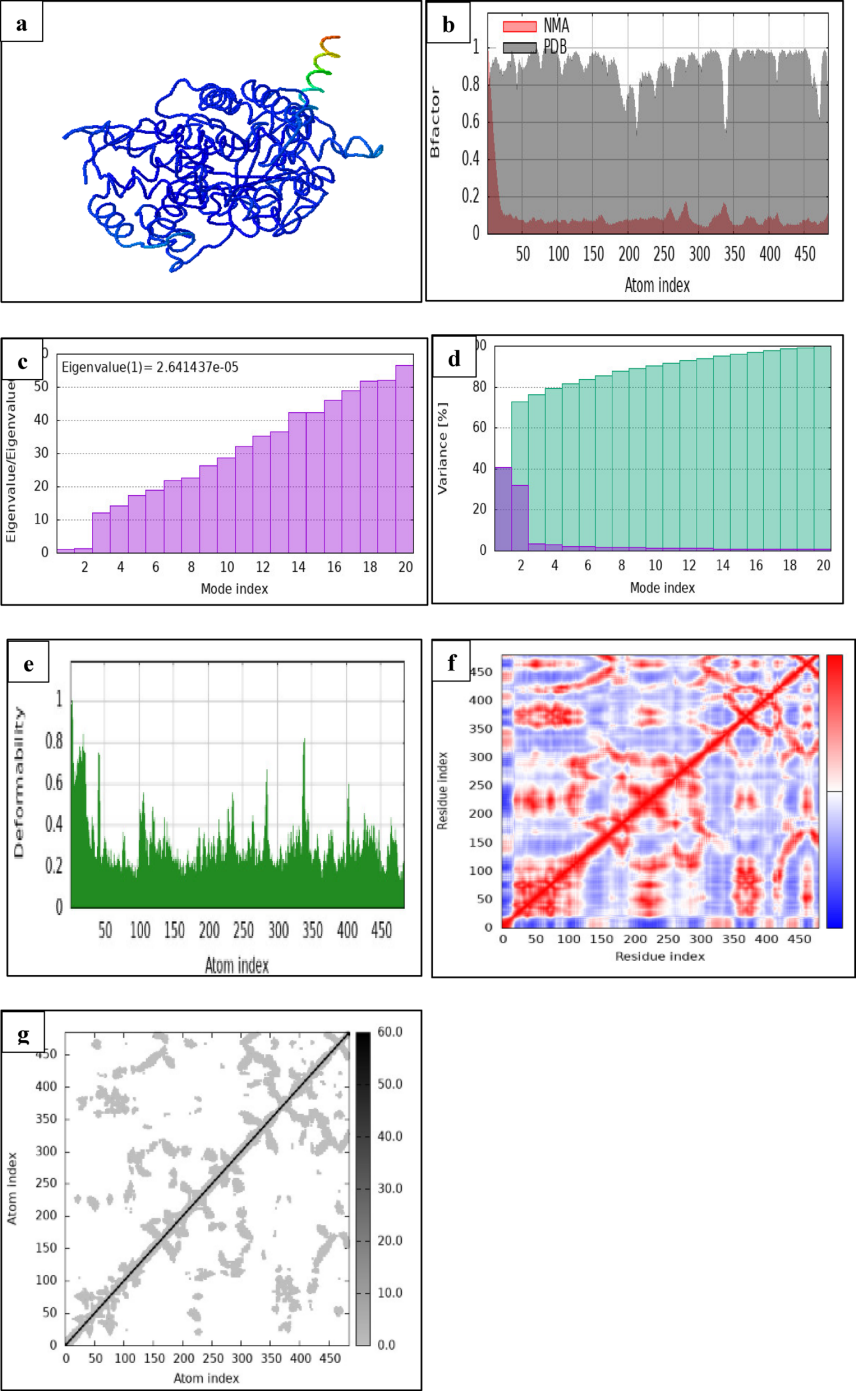
iMODS results for the interaction of 1-methyl-4-(1-methylethylidene)cyclohexene with Cytochrome P450 3A24. (**a**) 3D protein structure visualizing secondary structural elements in blue, with a green-to-red gradient highlighting a functionally significant region. (**b**) B-factor plot comparing atomic flexibility between Normal Mode Analysis (NMA) and Protein Data Bank (PDB) data. PDB shows higher B-factors, indicating greater atomic displacement under experimental conditions. (**c**) Eigenvalue graph showing the relationship between mode index and eigenvalue ratios. The gradual increase in eigenvalue ratio is key for dimensionality reduction and vibrational analysis, with an annotation indicating “Eigenvalue (1) = 2.641437e-05” for reference. (**d**) Variance plot illustrating individual and cumulative variance percentages across different mode indices. Purple bars indicate higher individual variance contributions, while green bars show cumulative accumulation, helping to identify dominant modes in structural dynamics. (**e**) Deformability plot highlighting atomic flexibility across indexed atoms. Peaks indicate higher flexibility, while troughs represent rigid segments, aiding in molecular behavior and dynamic analysis. (**f**) Co-variance map, residue-residue interaction heatmap, mapping protein connectivity through contact intensity. Red regions indicate strong residue interactions, while off-diagonal elements highlight structural folding and binding sites. (**g**) Elasticity map, atomic interaction heatmap, representing interatomic correlations, where color intensity reflects interaction strength. Diagonal trends indicate self-correlation, while the off-diagonal clusters highlight molecular stability and dynamic behavior.

To better understand the structural flexibility and interaction dynamics of 1-methyl-4-(1-methylethylidene)cyclohexene with two key detoxification enzymes—glutathione S-transferase theta 1–1 (GSTT1) and cytochrome P450 3A24 (CYP3A24)—a comprehensive molecular dynamics and normal mode analysis (NMA) was conducted. These analyses, illustrated in Figs. 6 and 7, provided insights into the dynamic behavior, atomic displacement, and flexibility of each protein–ligand complex under simulated physiological conditions.

Initial three-dimensional structural visualizations (Figs. 6a and 7a) show secondary structure elements highlighted in blue, with a green-to-red gradient indicating regions of potential functional relevance. These colored regions point to segments within each protein that may play a significant role in ligand binding or conformational adaptability. Notably, the red-shaded flexible domains suggest zones that could be more responsive to ligand-induced perturbations, which may affect enzymatic activity or substrate specificity.

To evaluate the flexibility of protein residues, B-factor comparisons (Figs. 6b and 7b) were made between theoretical NMA results and experimentally derived data from the Protein Data Bank (PDB). In both complexes, the PDB datasets showed generally higher B-factor values, reflecting greater atomic displacement under experimental conditions than predicted by NMA. This discrepancy is consistent with the intrinsic limitations of theoretical models in capturing environmental variability but also emphasizes critical regions of structural mobility—particularly in surface-exposed loops or ligand-binding clefts.

The eigenvalue plots (Figs. 6c and 7c) further complement this analysis by quantifying the energy required to deform each complex. GSTT1 displayed a higher first eigenvalue (2.675945e-04), suggesting greater structural stiffness, whereas CYP3A24 showed a markedly lower eigenvalue (2.641437e-05), indicative of more flexible global motions. These findings reflect the distinct functional roles of each enzyme: GSTT1 may benefit from rigid active sites to accommodate a broader range of substrates, while CYP3A24’s enhanced flexibility likely supports its monooxygenase catalytic mechanism.

Expanding on this, the variance plots (Figs. 6d and 7d) illustrate how motion is distributed across different vibrational modes. In both proteins, the early modes contributed disproportionately to atomic motion, with purple bars indicating individual variance and green bars representing cumulative variance. This pattern suggests that a small number of principal modes govern most of the dynamic behavior, underscoring the importance of low-frequency motions in structural adaptability and ligand responsiveness.

The deformability profiles (Figs. 6e and 7e) offer a more localized view of protein flexibility, mapping regions capable of significant structural rearrangement. GSTT1 exhibited several high deformability peaks, especially in loop regions, which may allow it to adjust more readily to ligand binding. In contrast, CYP3A24 showed relatively lower and more consistent deformability, aligning with its need for structural integrity in catalysis. These differences imply that while GSTT1 may accommodate diverse ligands through flexible binding domains, CYP3A24 relies on a tightly regulated binding conformation.

Supporting this observation, the residue–residue contact maps (Figs. 6f and 7f) highlight internal protein interactions critical for folding and stability. Strong red diagonal lines indicate self-interactions and backbone cohesion, while off-diagonal signals point to long-range residue interactions involved in maintaining tertiary structure. GSTT1 exhibited a broader interaction spectrum, potentially allowing for structural rearrangement upon ligand binding, whereas CYP3A24’s contact map revealed more localized and rigid interaction clusters, characteristic of a precise catalytic environment.

Finally, the atomic correlation heatmaps (Figs. 6g and 7g) provide a global overview of interatomic synchronization within each protein. Diagonal bands reflect internal self-correlations, while off-diagonal elements denote correlated or anticorrelated motions between spatially distinct regions. GSTT1 displayed broader anticorrelated areas, suggesting concerted movements that may support flexible domain communication. Conversely, CYP3A24 showed tighter clusters of correlated motion, reinforcing its structural rigidity and possibly explaining its substrate-specific action.

Together, these NMA and molecular dynamics results reveal a mechanistic contrast in the dynamic behavior of the two proteins. GSTT1 demonstrates higher flexibility and broader atomic coordination, potentially enhancing its ability to interact with diverse ligands and inhibiting detoxification pathways. On the other hand, CYP3A24’s constrained flexibility and localized correlated motions suggest a more specialized interaction with the ligand, targeting specific catalytic residues such as TYR418A. The ligand’s ability to engage with both flexible and rigid protein domains indicates a dual dynamic interaction strategy—one that allows conformational adaptation in GSTT1 and promotes stable binding in CYP3A24.

These findings provide a structural and biophysical basis for the observed inhibitory potential of 1-methyl-4-(1-methylethylidene) cyclohexene, a phytochemical constituent of *Punica granatum*. By exploiting both flexible and rigid regions within detoxification and metabolic enzymes of *Caligus clemensi*, the ligand may disrupt essential biochemical pathways. This dual-target flexibility, paired with its dynamic compatibility, supports the compound’s promise as an eco-friendly antiparasitic agent in aquaculture, warranting further in vivo and species-specific investigation.

## Discussion

Gas chromatography–mass spectrometry (GC–MS) analysis of *Punica granatum* methanolic peel extract revealed a chemically diverse profile dominated by monoterpenes, aromatic hydrocarbons, and fatty acid derivatives—classes known for potent antiparasitic, antimicrobial, and anti-inflammatory activities^40^. The phytochemical analysis conducted in this study provides critical insights into the antiparasitic mechanisms of *P. granatum* extract. The identified compounds, particularly the monoterpenes and aromatic hydrocarbons, likely contribute to the extract’s efficacy through multiple pathways including membrane disruption, neurotoxicity, and enzyme inhibition.

The extract also contained aromatic hydrocarbons such as *p*-cymene and *m*-cymene. While these compounds may exhibit limited standalone antiparasitic activity, their ability to enhance membrane fluidity and permeability can facilitate the uptake of more potent monoterpenes, thereby functioning as synergistic enhancers of overall extract efficacy^54,55^.

Fatty acid derivatives, including palmitic acid and monopalmitin, represent another key class of compounds in the extract. Saturated fatty acids like palmitic acid are known to influence membrane dynamics, potentially disrupting essential parasite functions. Monopalmitin, a monoester of palmitic acid, exhibits antimicrobial and antiparasitic effects, possibly by targeting surface lipids or disrupting biofilm formation. The identification of silylated derivatives of palmitic acid suggests enhanced chemical stability or bioavailability, further contributing to antiparasitic activity^47,48^. The combined action of these phytochemicals likely results in a synergistic mechanism that enhances antiparasitic efficacy. Monoterpenes primarily disrupt the nervous system and cellular membranes, while fatty acid derivatives modulate membrane structure and increase susceptibility to disruption. Aromatic hydrocarbons enhance the intracellular delivery of active compounds, amplifying their bioactivity. This multifaceted approach reduces the likelihood of resistance development and provides a strong basis for using plant-based agents in aquaculture^56^.

To complement the phytochemical findings, an in silico study was conducted on 1-methyl-4-(1-methylethylidene)cyclohexene, one of the major monoterpenes identified in the extract. We selected this compound for molecular docking analysis based on several criteria: it was one of the most abundant compounds in our GC-MS analysis, representing 5.27% of total extract; smaller, structurally defined compounds like this monoterpene are easier to model in silico compared to complex polyphenols or tannins abundant in pomegranate; and monoterpenes have established antiparasitic activities that align with our bioassay results. Targeting two critical detoxification enzymes in *C. clemensi*—glutathione S-transferase theta 1–1 (GSTT1) and cytochrome P450 3A24 (CYP3A24)—molecular docking demonstrated strong ligand binding, particularly at conserved residues TYR116A (GSTT1) and TYR418A (CYP3A24). Glutathione S-transferase theta 1–1 (GSTT1) is a crucial detoxification enzyme in parasitic crustaceans that catalyzes the conjugation of reduced glutathione to electrophilic xenobiotics^57,58^. This enzyme plays a vital role in protecting parasites against oxidative stress and facilitating the metabolism of toxic compounds, making it an attractive target for antiparasitic drug development^59,60^. Cytochrome P450 3A24 (CYP3A24) is a monooxygenase enzyme involved in Phase I detoxification reactions in *C. clemensi*^61,62^. This enzyme catalyzes the oxidation of various substrates including steroids, fatty acids, and xenobiotics. CYP3A24 is particularly important for parasite survival as it metabolizes host-derived compounds and potential toxins, contributing to drug resistance mechanisms^63,64^. These interactions likely impair enzyme activity by blocking substrate binding or inducing conformational changes.

Further molecular dynamics and normal mode analyses revealed contrasting structural behaviors of the two enzymes. GSTT1 exhibited greater flexibility, as shown by higher B-factors, eigenvalues, and deformability scores. This suggests that GSTT1 may undergo conformational adaptations upon ligand binding, making it more susceptible to dynamic disruption. In contrast, CYP3A24 showed a rigid structure with lower deformability and fewer domain motions. The stable interaction of the ligand with TYR418A in this rigid region suggests a mechanism by which enzymatic activity may be locked in an inactive state.

Elastic network and covariance analyses supported these findings, indicating that ligand binding disrupts essential domain motions in both enzymes, with GSTT1 showing broader anticorrelated motion patterns and CYP3A24 exhibiting localized suppression of functional movements. This dual interaction mechanism—engaging both flexible and rigid enzymatic regions—may be central to the compound’s inhibitory potential.

Conventional antiparasitic agents such as organophosphates and avermectins act via singular biochemical pathways and are increasingly associated with resistance and environmental concerns. In contrast, the *P. granatum* extract demonstrates a multimodal mechanism of action, involving neurotoxic, membrane-disrupting, and enzyme-inhibitory pathways. This diversity reduces the probability of resistance development and underscores its suitability as a sustainable alternative. Moreover, as a plant-derived product, the extract offers a lower ecological footprint, reduced bioaccumulation potential, and minimal risk to non-target species—traits that are increasingly important for sustainable aquaculture.

The findings of this study support the use of *P. granatum* extract as a botanical parasiticide in aquaculture. Recent comprehensive reviews have confirmed pomegranate’s diverse bioactive profile, including its antiparasitic properties^65,66^. Recent molecular studies have further elucidated the antimicrobial mechanisms of pomegranate constituents through advanced computational approaches^67^. These investigations have validated traditional therapeutic applications while revealing novel molecular targets for parasite control. Its broad-spectrum bioactivity, environmental safety, and low risk of resistance make it a strong candidate for integrated pest management strategies. Practical application could involve incorporation into medicated feeds or as bath treatments, with dosing regimens based on our LC50 and LC100 data. The biodegradability of plant-based compounds offers significant advantages over synthetic chemicals, though future studies should evaluate environmental persistence and breakdown products in aquaculture systems. However, to transition from experimental validation to field application, further research is essential. Future studies should focus on in vivo efficacy, toxicity assessments, formulation development, and environmental impact evaluations in real-world aquaculture systems. Long-term studies examining potential resistance development, effects on non-target aquatic organisms, and optimal delivery methods are particularly important for commercial implementation. Several limitations must be acknowledged. The study primarily employed in vitro assays and in silico modeling, highlighting the need for validation through field trials under real aquaculture conditions. Molecular docking focused on a single representative compound rather than investigating synergistic effects of multiple bioactive compounds. Comprehensive toxicological studies on non-target aquatic organisms are necessary to ensure environmental safety. Long-term resistance development potential requires monitoring under continuous exposure conditions. The crude methanolic extract needs formulation development for practical aquaculture applications, and economic feasibility assessment compared to conventional treatments is required. Despite these limitations, this research provides a solid foundation for developing *P. granatum* extract as a natural antiparasitic agent in sustainable aquaculture management. The integration of phytochemical profiling, in vitro bioassays, and in silico molecular modeling represents a comprehensive approach to understanding the mechanistic basis of natural product efficacy against aquatic parasites.

## Conclusion

This study demonstrates that *P. granatum* methanolic peel extract possesses a diverse array of bioactive compounds, predominantly monoterpenes, aromatic hydrocarbons, and fatty acid derivatives, which collectively contribute to its potent antiparasitic activity against *C. clemensi*. The multimodal mechanism involving membrane disruption, neurotoxicity, and enzymatic inhibition, supported by in silico analyses targeting detoxification enzymes GSTT1 and CYP3A24, highlights the extract’s potential as an effective and sustainable botanical parasiticide. Compared to conventional synthetic agents, *P. granatum* extract offers an eco-friendly alternative with a lower risk of resistance development and environmental toxicity.

## Supplementary Information

Below is the link to the electronic supplementary material.

Supplementary Material 1

## Data Availability

All data that support the findings of this study are available upon request from the corresponding author. The 18S rRNA gene sequences of Caligus clemensi derived from this study were submitted to the GenBank database and assigned accession number: OR563780 and PV955728-PV955732.

## Declarations

### ARRIVE guidelines

The study was conducted in compliance with the ARRIVE guidelines and all methods were conducted following relevant guidelines and regulations.
