# Supplementary material for: Punica granatum extract demonstrates antiparasitic effects against Caligus clemensi through in silico and in vitro studies
Source: Sci Rep. 2025 Oct 7;15:35005. doi: 10.1038/s41598-025-19529-y (PMC12504749; doi:10.1038/s41598-025-19529-y)

My GC-MS Report

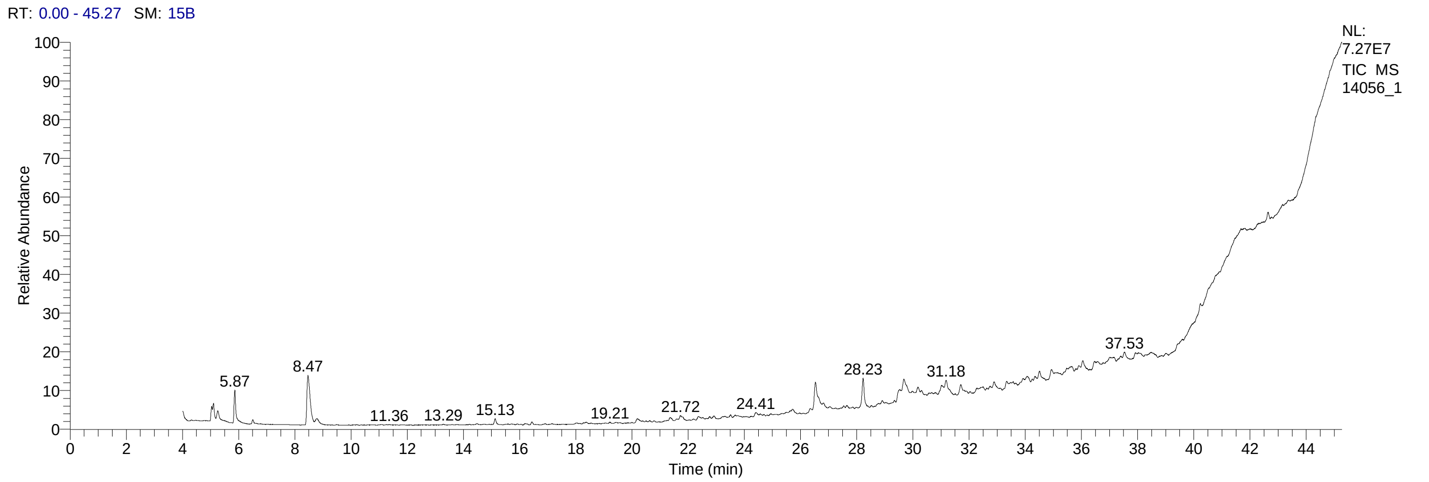

RT	Area %	Peak Area	Peak Height
5.04	5.27	7873536.08	3139607.26
5.10	6.54	9765225.47	3496029.40
5.86	15.03	22446345.30	7118779.91
8.46	34.46	51447965.90	8360697.50
26.53	21.13	31549810.32	4827238.90
28.23	17.57	26232116.23	5661007.28

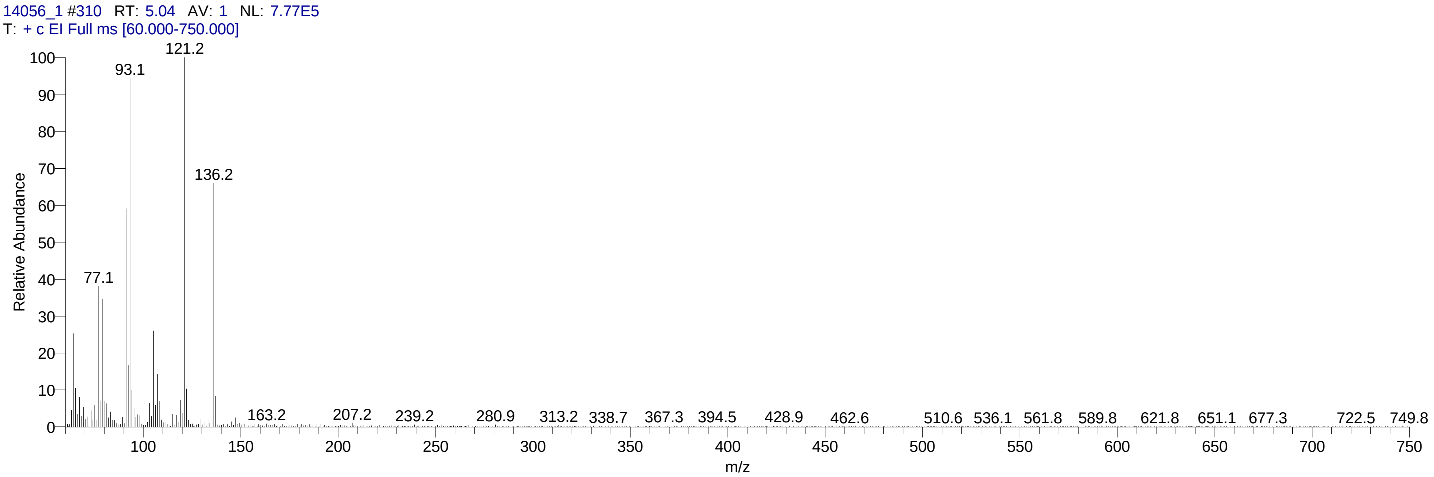

RT	Compound Name	Area %	MF	Molecular Formula	Molecular Weight	Cas #	Library
5.04	CYCLOHEXENE, 1-METHYL-4-(1-METHYLETHYLIDENE)-	5.27	899	C10H16	136	586-62-9	WileyRegistry8e
5.04	1,5,5-Trimethyl-6-methylene-cyclohexene	5.27	860	C10H16	136	514-95-4	mainlib
5.04	CYCLOHEXENE, 1,5,5-TRIMETHYL-6-METHYLENE-	5.27	860	C10H16	136	514-95-4	WileyRegistry8e
5.04	Cyclohexene, 1-methyl-4-(1-methylethylidene)-	5.27	886	C10H16	136	586-62-9	replib
5.04	2-Carene	5.27	900	C10H16	136	554-61-0	replib

My GC-MS Report

Compound Structure | Hit Spectrum

CYCLOHEXENE, 1-METHYL-4-(1-METHYLETHYLIDENE)-
Formula C10H16, MW 136, CAS# 586-62-9, Entry# 23371
1-METHYL-4-(1-METHYLETHYLIDENE)-1-CYCLOHEXENE #

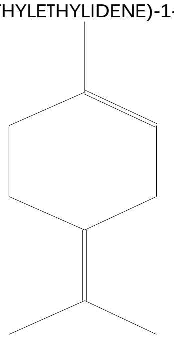

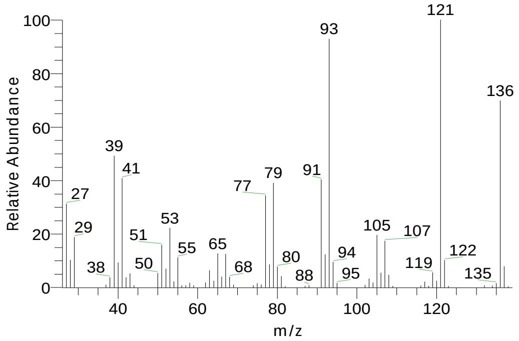

1,5,5-Trimethyl-6-methylene-cyclohexene
Formula C10H16, MW 136, CAS# 514-95-4, Entry# 102838
1,5,5-Trimethyl-6-methylene-1-cyclohexene #

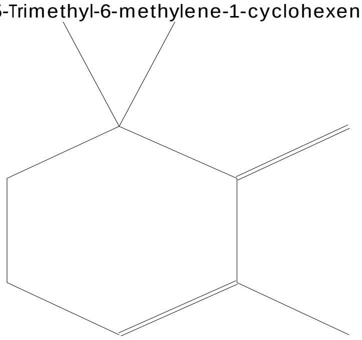

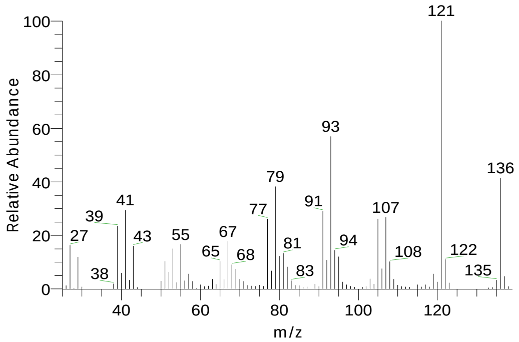

CYCLOHEXENE, 1,5,5-TRIMETHYL-6-METHYLENE-
Formula C10H16, MW 136, CAS# 514-95-4, Entry# 371135
1,5,5-TRIMETHYL-6-METHYLENE-CYCLOHEXENE

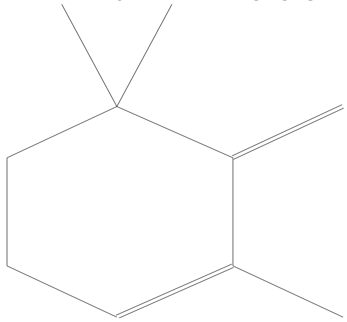

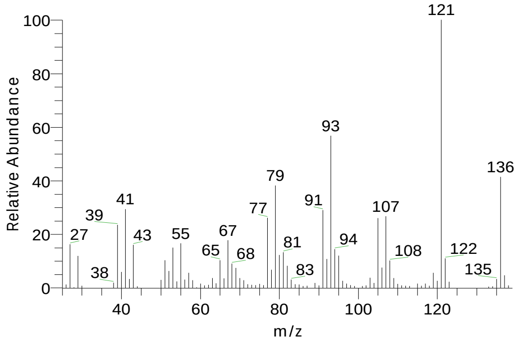

Cyclohexene, 1-methyl-4-(1-methylethylidene)-
Formula C10H16, MW 136, CAS# 586-62-9, Entry# 19183
p-Mentha-1,4(8)-diene

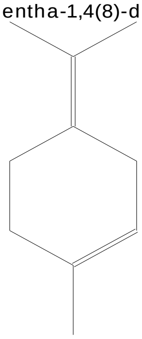

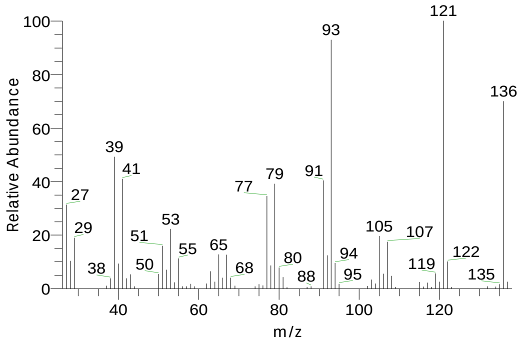

My GC-MS Report

Compound Structure | Hit Spectrum

2-Carene
Formula C10H16, MW 136, CAS# 554-61-0, Entry# 14191
Bicyclo[4.1.0]hept-2-ene, 3,7,7-trimethyl-

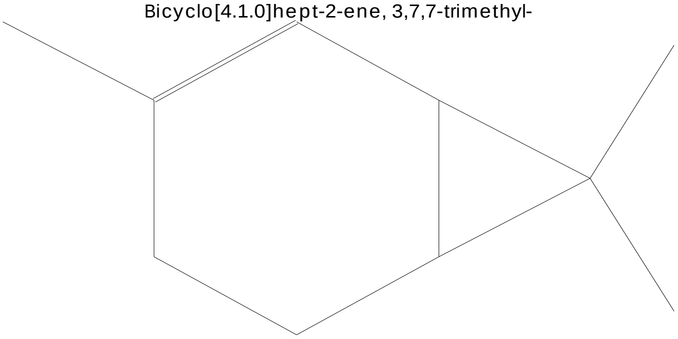

SI 806, RSI 900, replib, Entry# 14191, CAS# 554-61-0, 2-Carene

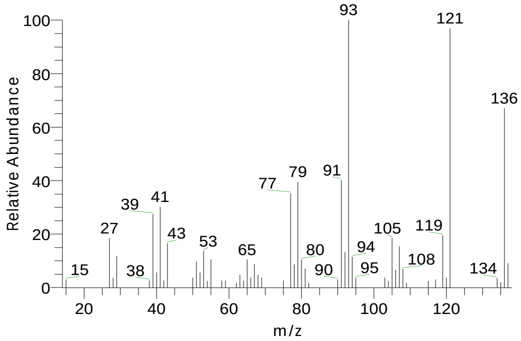

14056_1 #329 RT: 5.10 AV: 1 NL: 1.64E6
T: + c EI Full ms [60.000-750.000]

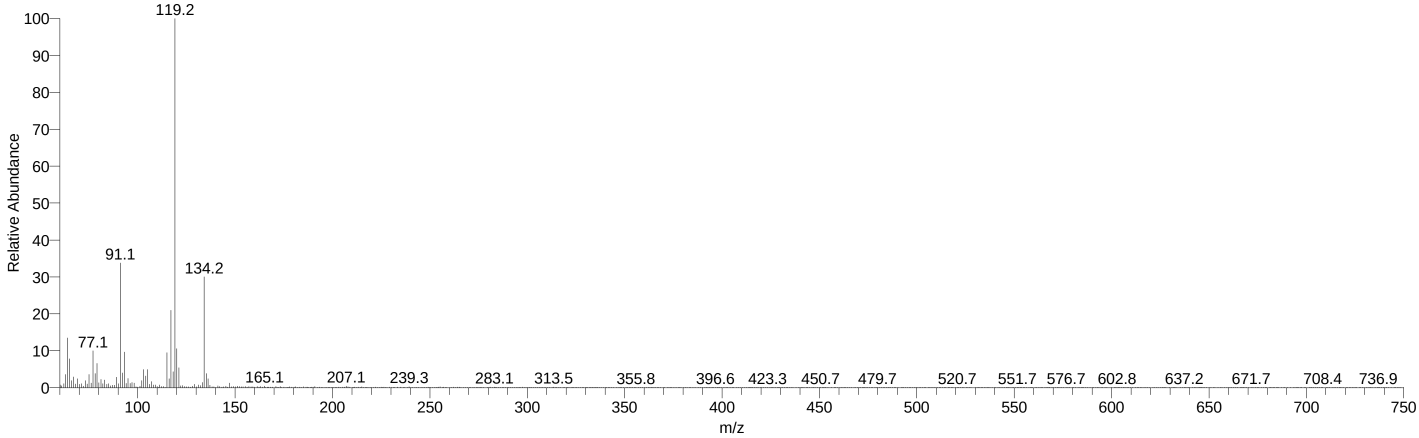

RT	Compound Name	Area %	MF	Molecular Formula	Molecular Weight	Cas #	Library
5.10	p-Cymene	6.54	842	C10H14	134	99-87-6	CaymanSpectralLibrary-NIST.HP
5.10	BENZENE, 1-METHYL-3-(1-METHYLETHYL)-	6.54	868	C10H14	134	535-77-3	WileyRegistry8e
5.10	p-Cymene	6.54	839	C10H14	134	99-87-6	mainlib
5.10	BENZENE, 1-METHYL-4-(1-METHYLETHYL)-	6.54	838	C10H14	134	99-87-6	WileyRegistry8e
5.10	BENZENE, 1-METHYL-4-(1-METHYLETHYL)-	6.54	863	C10H14	134	99-87-6	WileyRegistry8e

Compound Structure | Hit Spectrum

p-Cymene
Formula C10H14, MW 134, CAS# 99-87-6, Entry# 1400
Benzene, 1-methyl-4-(1-methylethyl)-

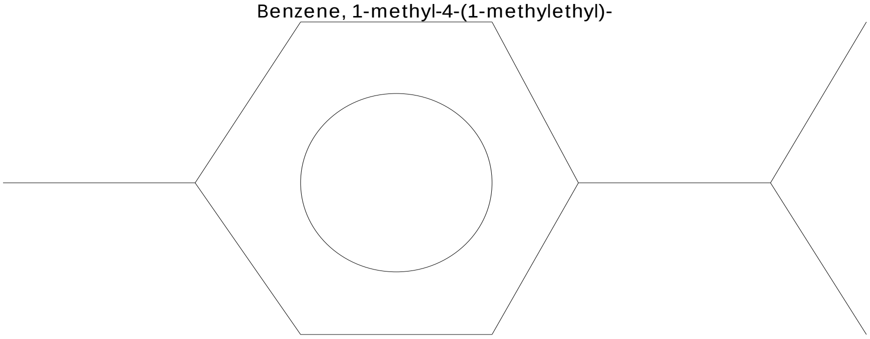

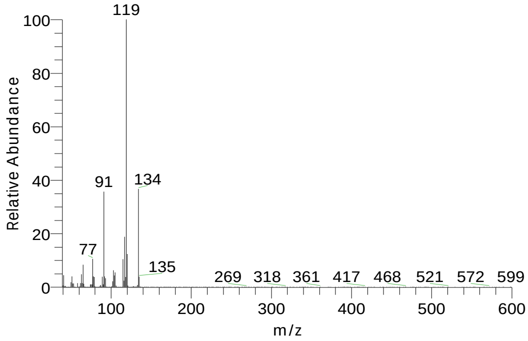

My GC-MS Report

Compound Structure | Hit Spectrum

BENZENE, 1-METHYL-3-(1-METHYLETHYL)-
Formula C10H14, MW 134, CAS# 535-77-3, Entry# 21767
M-CYMENE

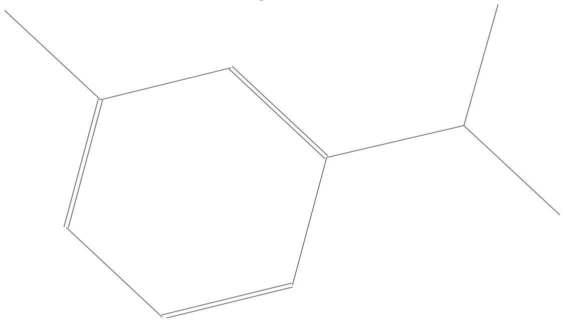

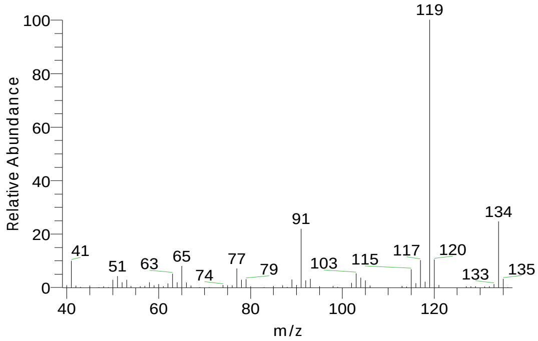

SI 816, RSI 839, mainlib, Entry# 100331, CAS# 99-87-6, p-Cymene

p-Cymene
Formula C10H14, MW 134, CAS# 99-87-6, Entry# 100331
Benzene, 1-methyl-4-(1-methylethyl)-

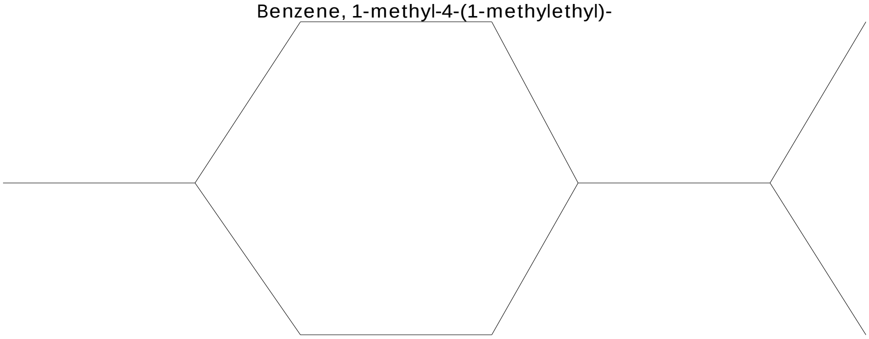

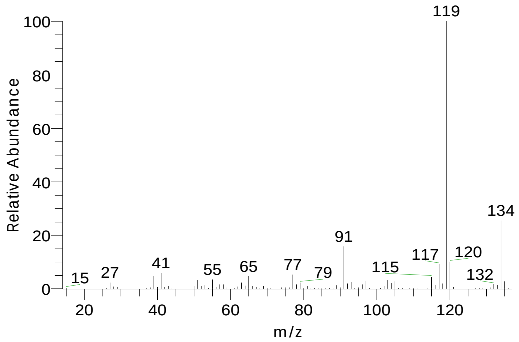

BENZENE, 1-METHYL-4-(1-METHYLETHYL)-
Formula C10H14, MW 134, CAS# 99-87-6, Entry# 21775
P-CYMENE

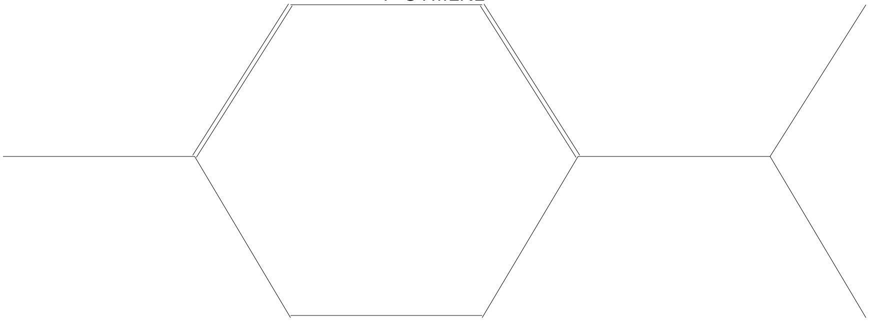

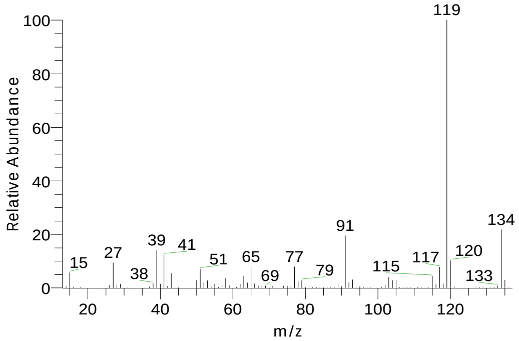

BENZENE, 1-METHYL-4-(1-METHYLETHYL)-
Formula C10H14, MW 134, CAS# 99-87-6, Entry# 21777
P-CYMENE

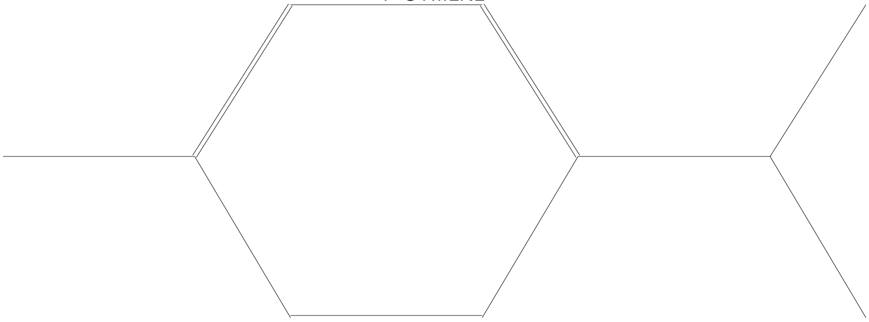

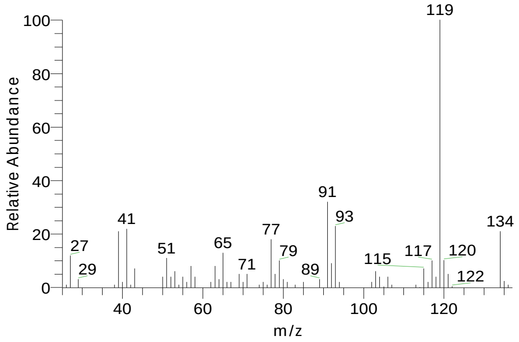

My GC-MS Report

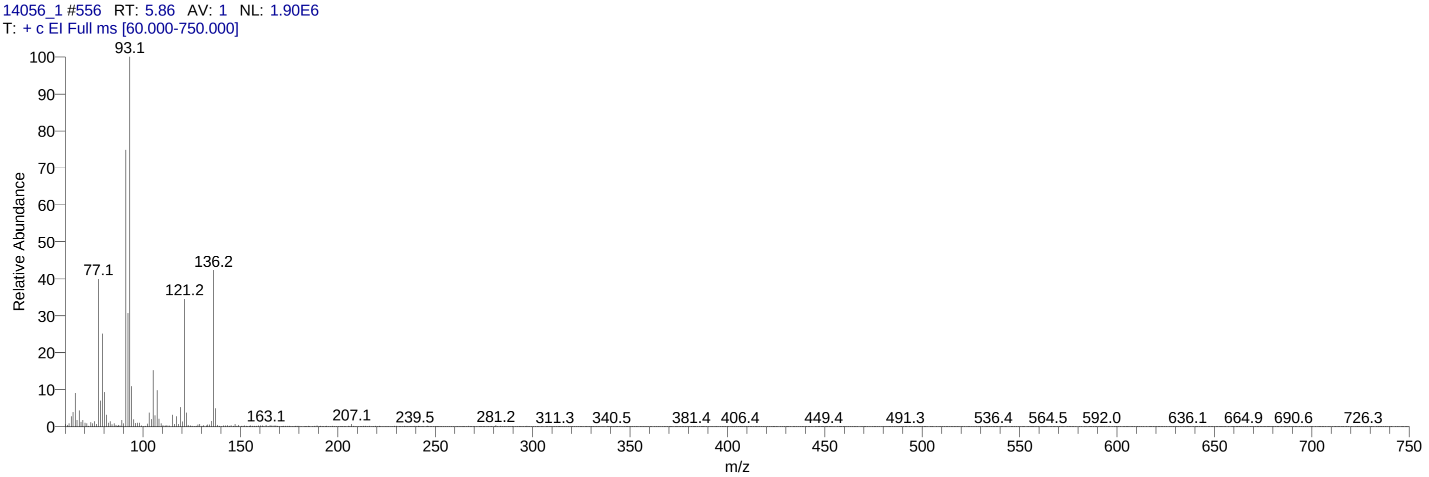

RT	Compound Name	Area %	MF	Molecular Formula	Molecular Weight	Cas #	Library
5.86	ç-Terpinene	15.03	938	C10H16	136	99-85-4	replib
5.86	ç-Terpinene	15.03	913	C10H16	136	99-85-4	replib
5.86	1,4-CYCLOHEXADIENE, 1-METHYL-4-(1-METHYLETHYL)-	15.03	913	C10H16	136	99-85-4	WileyRegistry8e
5.86	ç-Terpinene	15.03	914	C10H16	136	99-85-4	replib
5.86	1,4-CYCLOHEXADIENE, 1-METHYL-4-(1-METHYLETHYL)-	15.03	906	C10H16	136	99-85-4	WileyRegistry8e

Compound Structure

Hit Spectrum

ç-Terpinene
Formula C10H16, MW 136, CAS# 99-85-4, Entry# 14110
1,4-Cyclohexadiene, 1-methyl-4-(1-methylethyl)-

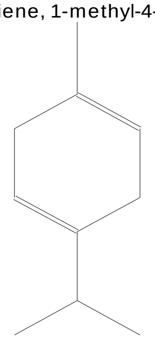

SI 901, RSI 938, replib, Entry# 14110, CAS# 99-85-4, ç-Terpinene

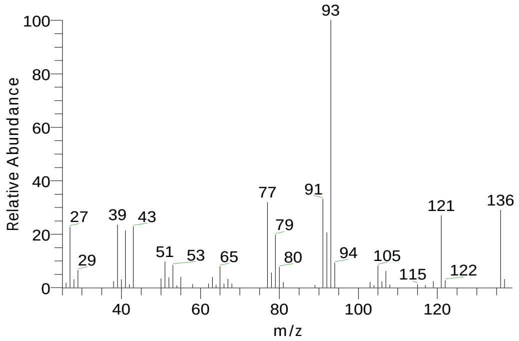

ç-Terpinene
Formula C10H16, MW 136, CAS# 99-85-4, Entry# 14112
1,4-Cyclohexadiene, 1-methyl-4-(1-methylethyl)-

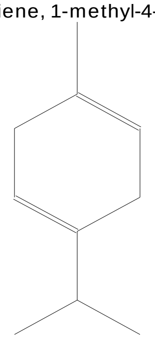

SI 893, RSI 913, replib, Entry# 14112, CAS# 99-85-4, ç-Terpinene

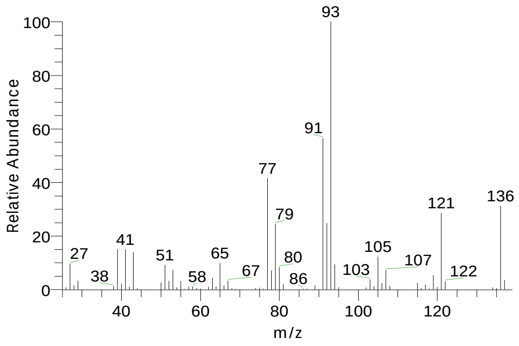

My GC-MS Report

Compound Structure | Hit Spectrum

1,4-CYCLOHEXADIENE, 1-METHYL-4-(1-METHYLETHYL)-
Formula C10H16, MW 136, CAS# 99-85-4, Entry# 380287
1-ISOPROPYL-4-METHYL-1,4-CYCLOHEXADIENE #

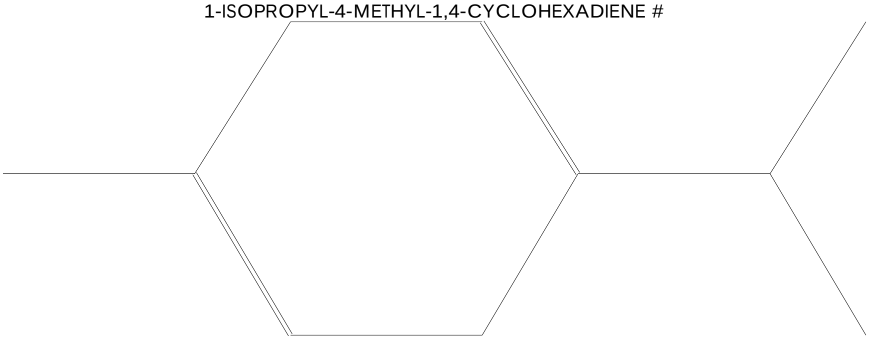

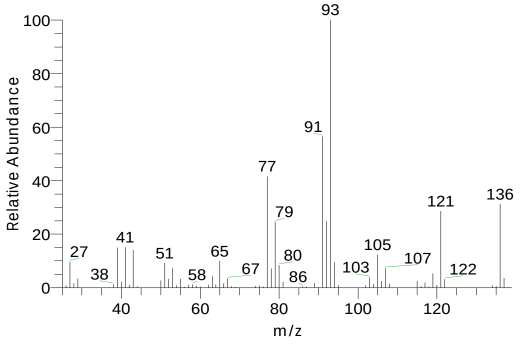

SI 892, RSI 914, replib, Entry# 14124, CAS# 99-85-4, ç-Terpinene

ç-Terpinene
Formula C10H16, MW 136, CAS# 99-85-4, Entry# 14124
1,4-Cyclohexadiene, 1-methyl-4-(1-methylethyl)-

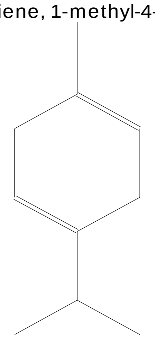

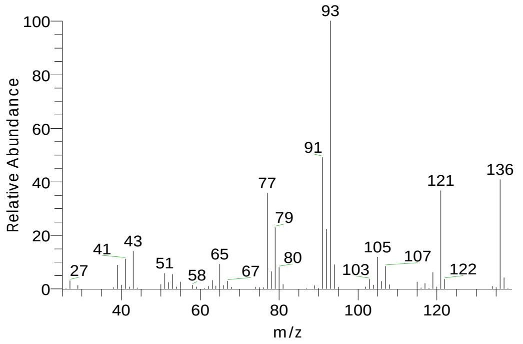

1,4-CYCLOHEXADIENE, 1-METHYL-4-(1-METHYLETHYL)-
Formula C10H16, MW 136, CAS# 99-85-4, Entry# 23301
1-ISOPROPYL-4-METHYL-1,4-CYCLOHEXADIENE #

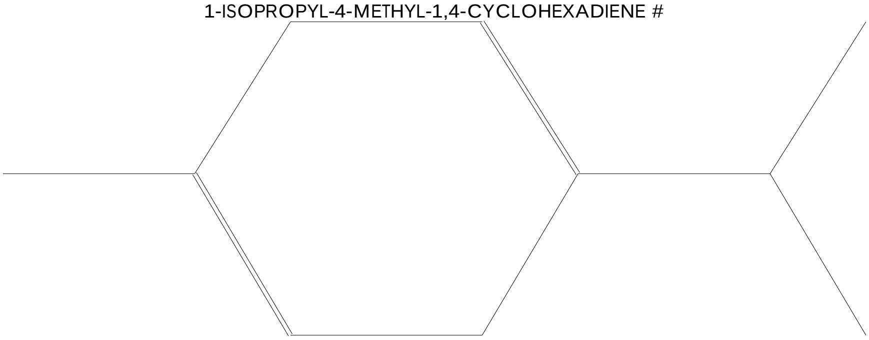

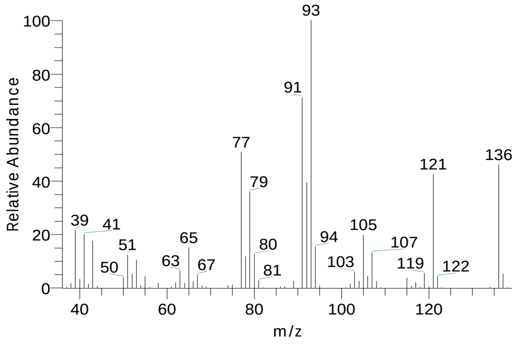

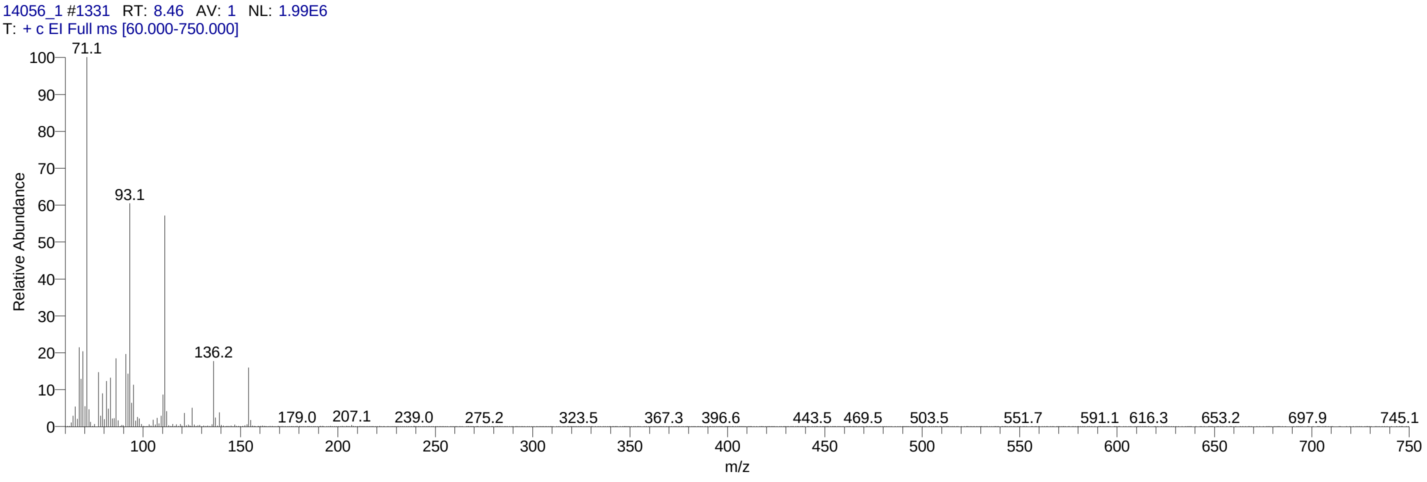

RT	Compound Name	Area %	MF	Molecular Formula	Molecular Weight	Cas #	Library
8.46	3-CYCLOHEXEN-1-OL, 4-METHYL-1-(1-METHYLETHYL)-	34.46	925	C10H18O	154	562-74-3	WileyRegistry8e

My GC-MS Report

RT	Compound Name	Area %	MF	Molecular Formula	Molecular Weight	Cas #	Library
8.46	3-CYCLOHEXEN-1-OL, 4-METHYL-1-(1-METHYLETHYL)-	34.46	933	C10H18O	154	562-74-3	WileyRegistry8e
8.46	3-CYCLOHEXEN-1-OL, 4-METHYL-1-(1-METHYLETHYL)-	34.46	932	C10H18O	154	562-74-3	WileyRegistry8e
8.46	3-CYCLOHEXEN-1-OL, 4-METHYL-1-(1-METHYLETHYL)-	34.46	917	C10H18O	154	562-74-3	WileyRegistry8e
8.46	3-Cyclohexen-1-ol, 4-methyl-1-(1-methylethyl)-, (R)-	34.46	928	C10H18O	154	20126-76-5	replib

Compound Structure | Hit Spectrum

3-CYCLOHEXEN-1-OL, 4-METHYL-1-(1-METHYLETHYL)-
Formula C10H18O, MW 154, CAS# 562-74-3, Entry# 38346
1-ISOPROPYL-4-METHYL-3-CYCLOHEXEN-1-OL #

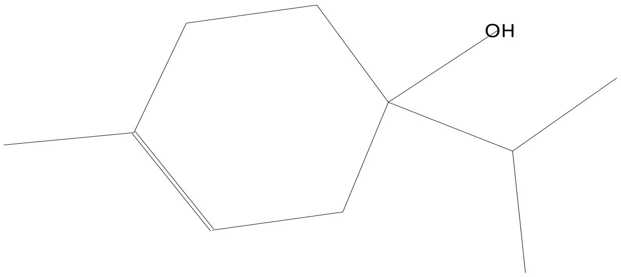

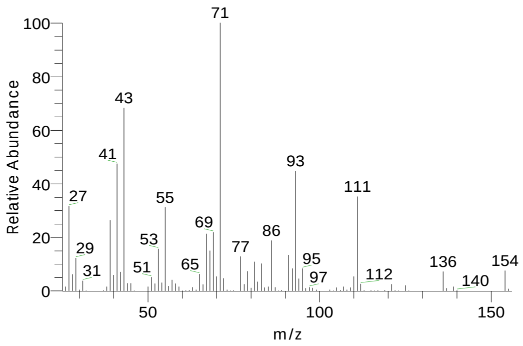

3-CYCLOHEXEN-1-OL, 4-METHYL-1-(1-METHYLETHYL)-
Formula C10H18O, MW 154, CAS# 562-74-3, Entry# 38350
1-ISOPROPYL-4-METHYL-3-CYCLOHEXEN-1-OL #

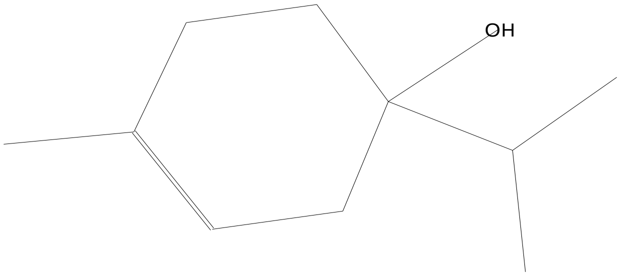

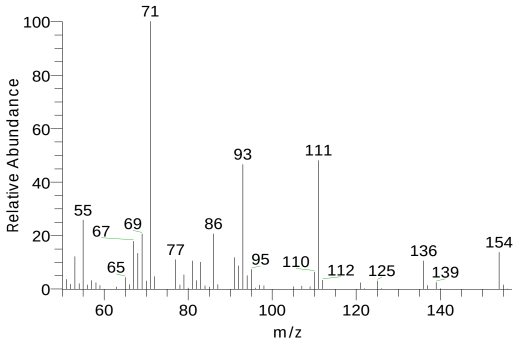

3-CYCLOHEXEN-1-OL, 4-METHYL-1-(1-METHYLETHYL)-
Formula C10H18O, MW 154, CAS# 562-74-3, Entry# 38353
1-ISOPROPYL-4-METHYL-3-CYCLOHEXEN-1-OL #

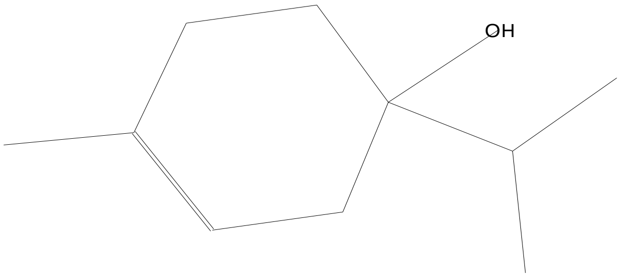

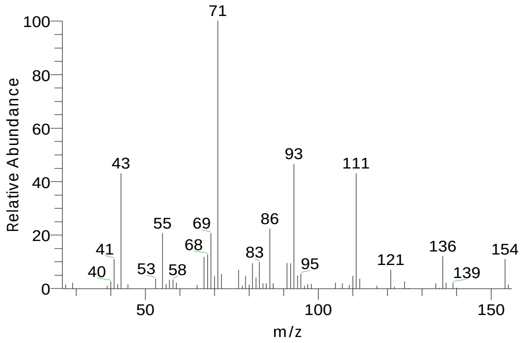

My GC-MS Report

Compound Structure	Hit Spectrum

3-CYCLOHEXEN-1-OL, 4-METHYL-1-(1-METHYLETHYL)-
Formula C10H18O, MW 154, CAS# 562-74-3, Entry# 38345
1-ISOPROPYL-4-METHYL-3-CYCLOHEXEN-1-OL #

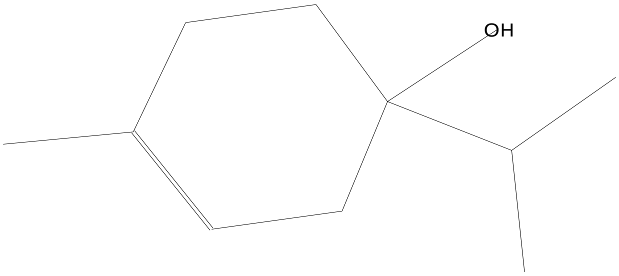

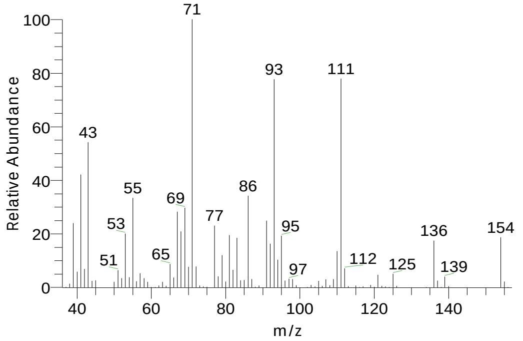

3-Cyclohexen-1-ol, 4-methyl-1-(1-methylethyl)-, (R)-
Formula C10H18O, MW 154, CAS# 20126-76-5, Entry# 9252
p-Menth-1-en-4-ol, (R)-(-)-

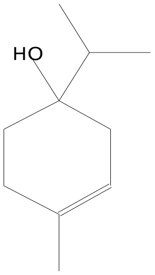

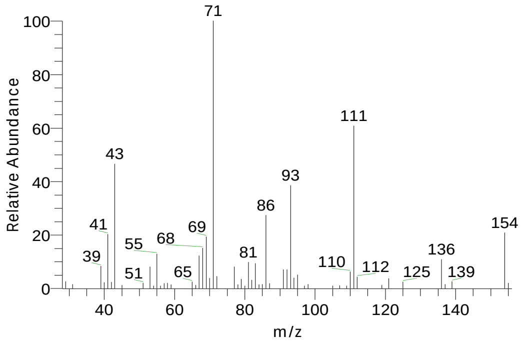

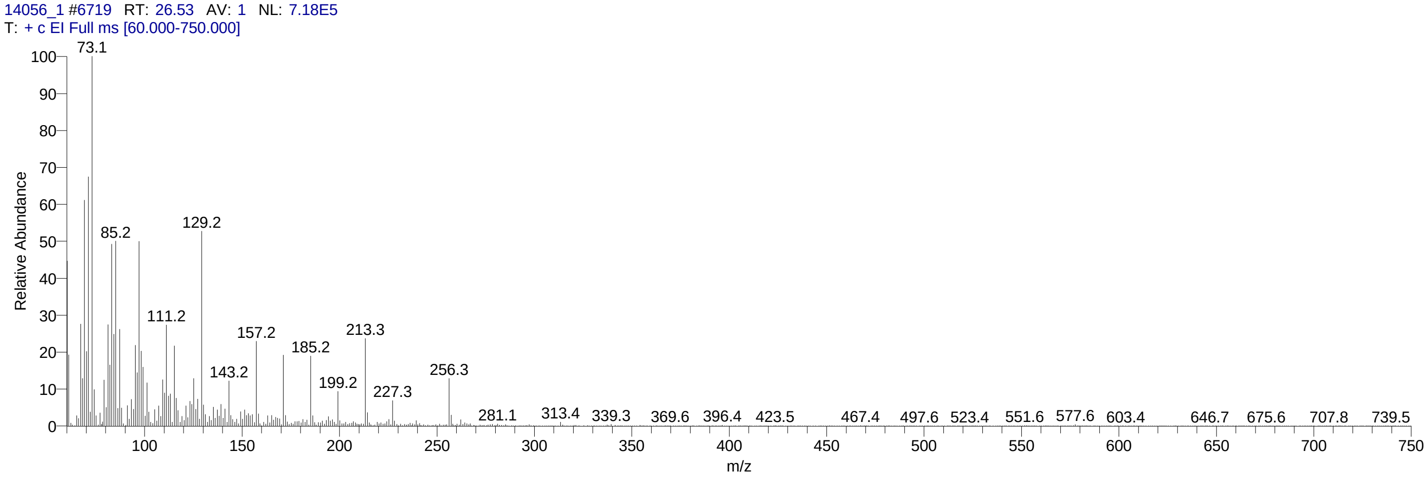

RT	Compound Name	Area %	MF	Molecular Formula	Molecular Weight	Cas #	Library
26.53	HEXADECANOIC ACID	21.13	864	C16H32O2	256	57-10-3	WileyRegistry8e
26.53	HEXADECANOIC ACID	21.13	853	C16H32O2	256	57-10-3	WileyRegistry8e
26.53	n-Hexadecanoic acid	21.13	859	C16H32O2	256	57-10-3	replib
26.53	HEXADECANOIC ACID	21.13	821	C16H32O2	256	57-10-3	WileyRegistry8e
26.53	HEXADECANOIC ACID, 2,3-DIHYDROXYPROPYL ESTER	21.13	810	C19H38O4	330	542-44-9	WileyRegistry8e

My GC-MS Report

Compound Structure | Hit Spectrum

HEXADECANOIC ACID
Formula C16H32O2, MW 256, CAS# 57-10-3, Entry# 397116
HEXADECANOATE

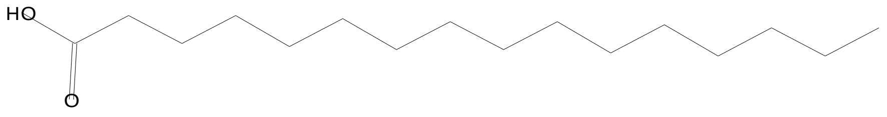

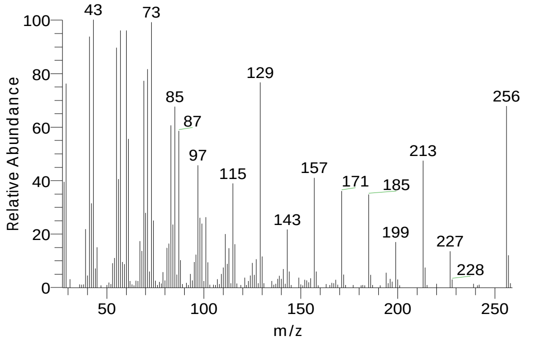

HEXADECANOIC ACID
Formula C16H32O2, MW 256, CAS# 57-10-3, Entry# 146744
HEXADECANOATE

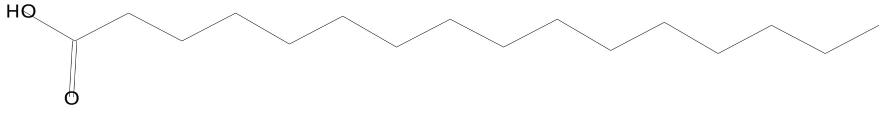

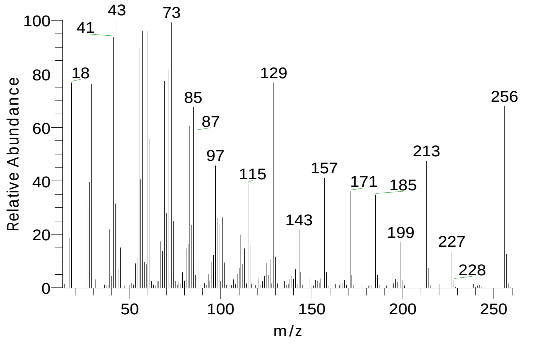

n-Hexadecanoic acid
Formula C16H32O2, MW 256, CAS# 57-10-3, Entry# 7566
Hexadecanoic acid

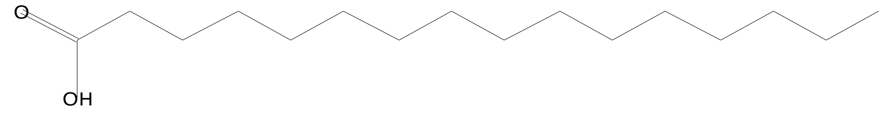

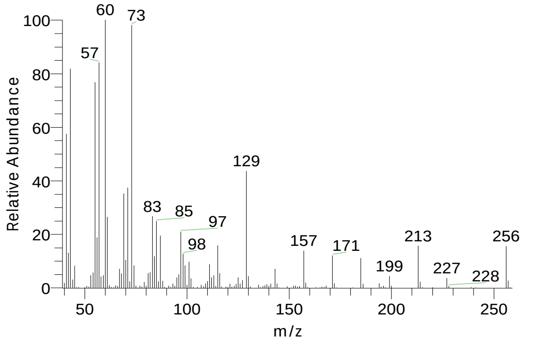

HEXADECANOIC ACID
Formula C16H32O2, MW 256, CAS# 57-10-3, Entry# 146751
HEXADECANOATE

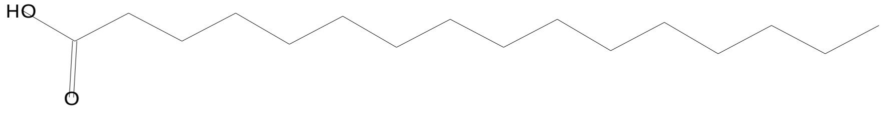

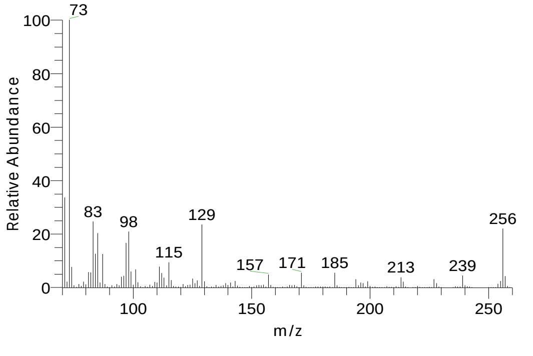

My GC-MS Report

Compound Structure | Hit Spectrum

HEXADECANOIC ACID, 2,3-DIHYDROXYPROPYL ESTER
Formula C19H38O4, MW 330, CAS# 542-44-9, Entry# 214589
2,3-DIHYDROXYPROPYL PALMITATE #

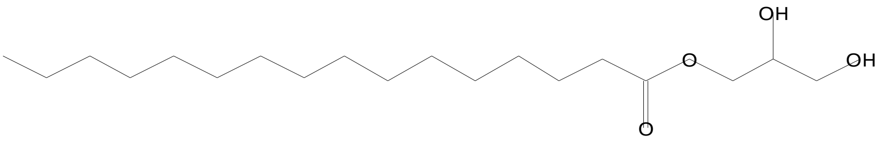

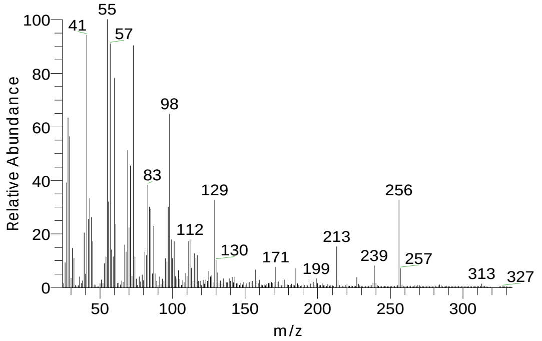

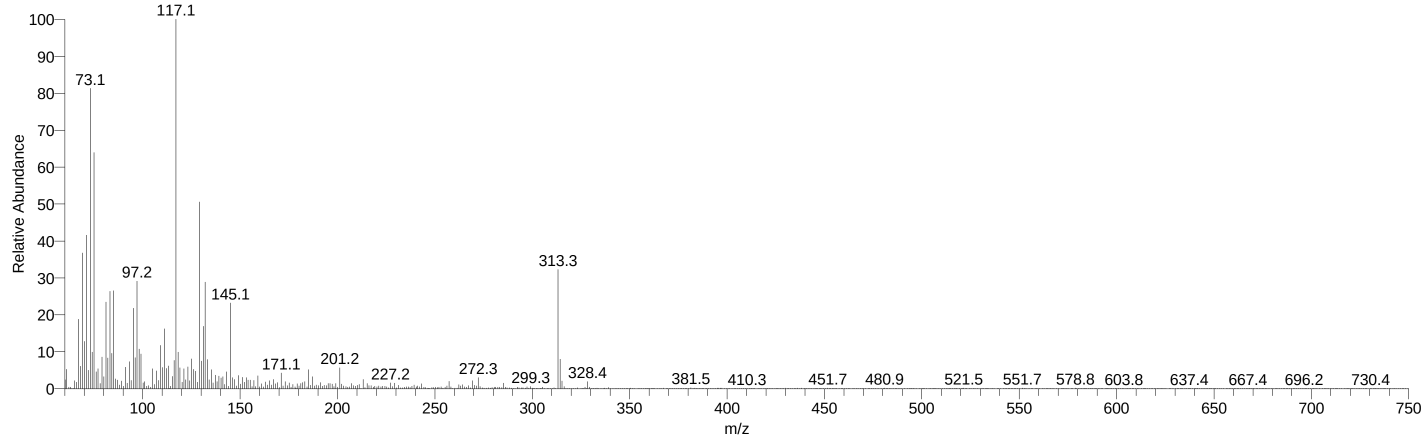

RT	Compound Name	Area %	MF	Molecular Formula	Molecular Weight	Cas #	Library
28.23	Palmitic Acid, TMS derivative	17.57	792	C19H40O2Si	328	55520-89-3	replib
28.23	HEXADECANOIC ACID, TRIMETHYLSILYL ESTER	17.57	792	C19H40O2Si	328	55520-89-3	WileyRegistry8e
28.23	HEXADECANOIC ACID, TRIMETHYLSILYL ESTER	17.57	791	C19H40O2Si	328	55520-89-3	WileyRegistry8e
28.23	Palmitic Acid, TMS derivative	17.57	774	C19H40O2Si	328	55520-89-3	mainlib
28.23	HEXADECANOIC ACID, TRIMETHYLSILYL ESTER	17.57	823	C19H40O2Si	328	55520-89-3	WileyRegistry8e

Compound Structure | Hit Spectrum

Palmitic Acid, TMS derivative
Formula C19H40O2Si, MW 328, CAS# 55520-89-3, Entry# 9711
Hexadecanoic acid, trimethylsilyl ester

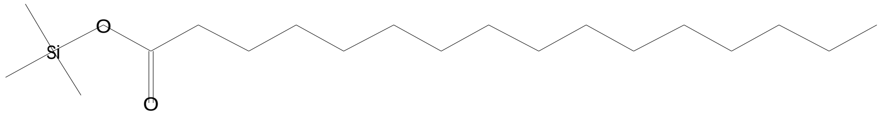

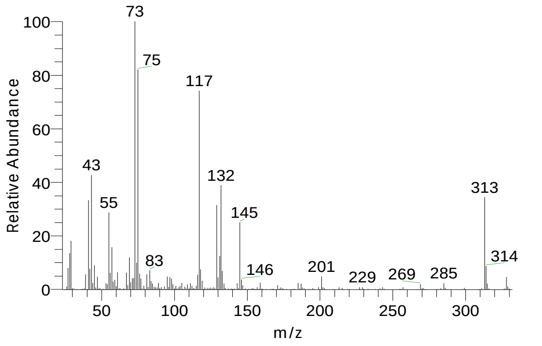

My GC-MS Report

Compound Structure | Hit Spectrum

HEXADECANOIC ACID, TRIMETHYLSILYL ESTER
Formula C19H40O2Si, MW 328, CAS# 55520-89-3, Entry# 390490
TRIMETHYLSILYL PALMITATE #

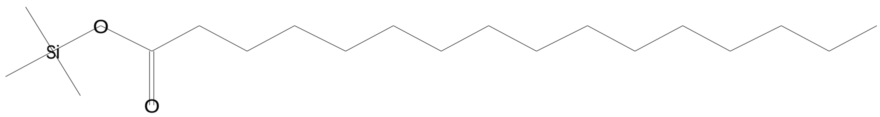

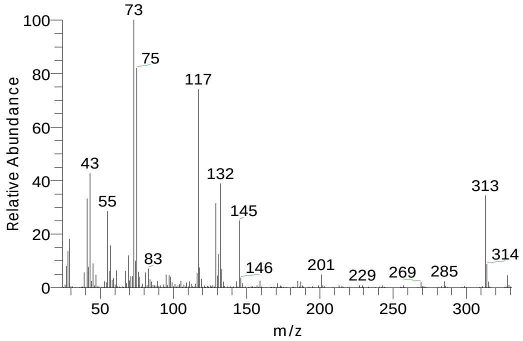

HEXADECANOIC ACID, TRIMETHYLSILYL ESTER
Formula C19H40O2Si, MW 328, CAS# 55520-89-3, Entry# 213028
TRIMETHYLSILYL PALMITATE #

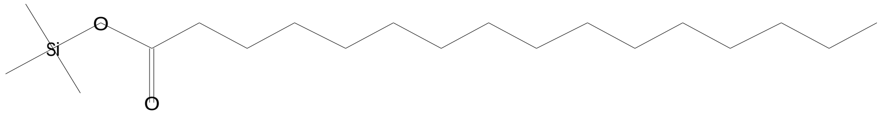

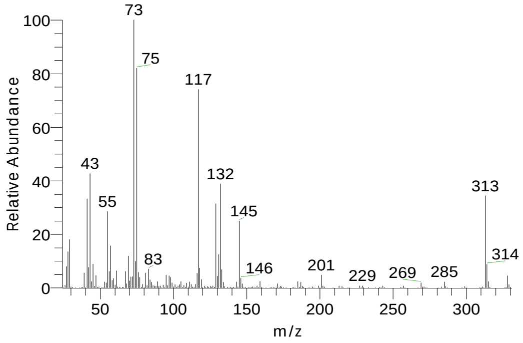

Palmitic Acid, TMS derivative
Formula C19H40O2Si, MW 328, CAS# 55520-89-3, Entry# 97113
Hexadecanoic acid, trimethylsilyl ester

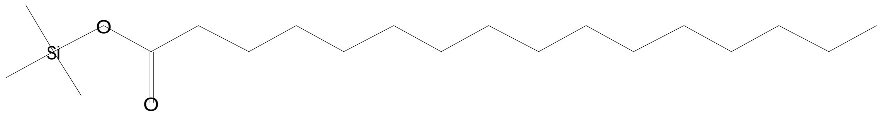

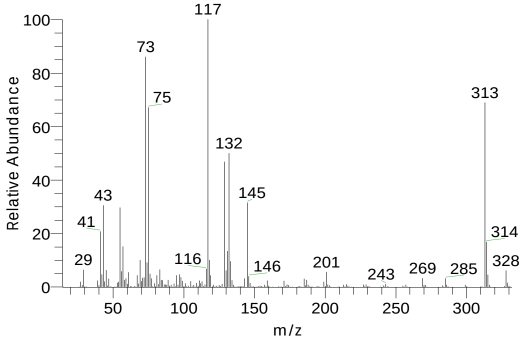

HEXADECANOIC ACID, TRIMETHYLSILYL ESTER
Formula C19H40O2Si, MW 328, CAS# 55520-89-3, Entry# 213031
TRIMETHYLSILYL PALMITATE #

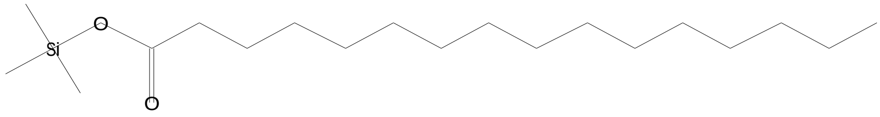

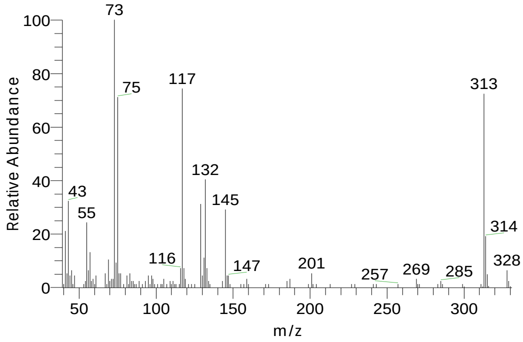

Supplement: Supplementary file 1 — Supplementary Material 1 [file 41598_2025_19529_MOESM1_ESM.pdf]
